# ARNT deficiency represses pyruvate dehydrogenase kinase 1 to trigger ROS production and melanoma metastasis

**DOI:** 10.1038/s41389-020-00299-3

**Published:** 2021-01-14

**Authors:** Chi-Ruei Huang, Ting-Wei Chang, Chung-Ta Lee, Chih-Jie Shen, Wen-Chang Chang, Ben-Kuen Chen

**Affiliations:** 1 Department of Biotechnology and Bioindustry Sciences, College of Bioscience and Biotechnology, National Cheng Kung University, Tainan, 701 Taiwan; 2 Graduate Institute of Medical Sciences, College of Medicine, Taipei Medical University, Taipei, 110 Taiwan; 3 Institute of Basic Medical Sciences, College of Medicine, National Cheng Kung University, Tainan, 701 Taiwan; 4 Department of Pathology, National Cheng Kung University Hospital, College of Medicine, National Cheng Kung university, Tainan, 701 Taiwan; 5 Department of Pharmacology, College of Medicine, National Cheng Kung University, Tainan, 701 Taiwan; 6 Institute for Cancer Biology and Drug Discovery, College of Medical Science and Technology, Taipei Medical University, Taipei, 110 Taiwan

**Keywords:** Melanoma, Metastasis

## Abstract

The metabolic changes in melanoma cells that are required for tumor metastasis have not been fully elucidated. In this study, we show that the increase in glucose uptake and mitochondrial oxidative phosphorylation confers metastatic ability as a result of aryl hydrocarbon receptor nuclear translocator (ARNT) deficiency. In clinical tissue specimens, increased ARNT, pyruvate dehydrogenase kinase 1 (PDK1), and NAD(P)H quinine oxidoreductase-1 (NQO1) was observed in benign nevi, whereas lower expression was observed in melanoma. The depletion of ARNT dramatically repressed PDK1 and NQO1 expression, which resulted in an increase of ROS levels. The elimination of ROS using N-acetylcysteine (NAC) and inhibition of oxidative phosphorylation using carbonyl cyanide m-chlorophenyl hydrazone (CCCP) and rotenone inhibited the ARNT and PDK1 deficiency-induced cell migration and invasion. In addition, ARNT deficiency in tumor cells manipulated the glycolytic pathway through enhancement of the glucose uptake rate, which reduced glucose dependence. Intriguingly, CCCP and NAC dramatically inhibited ARNT and PDK1 deficiency-induced tumor cell extravasation in mouse models. Our work demonstrates that downregulation of ARNT and PDK1 expression serves as a prognosticator, which confers metastatic potential as the metastasizing cells depend on metabolic changes.

## Introduction

Melanoma progression occurs as a result of the malignant proliferation of melanocytes that metastasisize, which contains a series of steps including the following: benign precursor lesion formation (benign nevus), dysplastic nevi formation, radial-growth and vertical-growth phase, and ultimately metastasis^1^. More than 95% of melanoma patients with multiple sites of metastatic disease die within 1 year^2^. The accumulation of genetic and epigenetic changes including BRAF and NRAS mutations is thought to drive melanoma progression^3^. For example, BRAF V600E/K genetic alteration is found in approximately 50% of all melanomas^4^. In addition, aryl hydrocarbon receptor (AhR), which is a member of the basic helix-loop-helix/PER-ARNT-SIM (bHLH-PAS) family promotes the tumorigenicity of melanoma^5,6^. Intriguingly, recent reports show that activation of AhR has been associated with resistance to BRAF-inhibitors and tumor dormancy in melanoma^7–9^.

The aryl hydrocarbon receptor nuclear translocator (ARNT) belongs to the bHLH-PAS family of transcription factors^10^. As a multifunctional protein, ARNT interacts with other bHLH/PAS members to drive different cellular functions^11^. For example, the HIF1α/ARNT complex promotes tumor angiogenesis, erythropoiesis and glycolysis^12^. The AhR/ARNT heterodimer upregulates cytochrome P450 expression which is associated with melanoma prognosis^13,14^. The ARNT/Sp1/c-Jun complex also regulates MDR1 expression and EGF-induced gene expression, such as that of cyclooxygenase-2, p21^WAF1/CIP1^ and 12(S)-lipoxygenase, which contribute to cancer drug resistance and tumor metastasis^15–17^. Although the molecular mechanisms involved in bHLH-PAS family-regulated tumor progression and metastasis have become better understood in recent years, the precise role of ARNT in tumorigenesis is still unclear. Through its interaction with HIF-1α, ARNT has been recognized as an oncoprotein that promotes tumor growth in response to hypoxia^18^. On the contrary, our previous studies show that decreased expression of ARNT by miR-107 targeting enhances tumor metastasis^19^. These results suggest that ARNT may enhance tumor growth in early-stage tumors but inhibit tumor metastasis in late-stage tumors. Nevertheless, the molecular mechanisms underlying the functional association between ARNT downregulation and tumor metastasis such as in melanoma remain unclear.

The glycolytic switch is an important phenomenon in the metabolic regulation of cancer progression. For example, liver-specific metastatic breast cancer cells predominantly use the HIF-1α/pyruvate dehydrogenase kinase 1 (PDK1) pathway to maintain a glycolytic phenotype^20^. An increase in hexokinase 2 (HK2) via EGFR activation contributes to aerobic glycolysis in triple-negative breast cancer cells^21^. In addition, the combined treatment with BRAF and PDK1 inhibitors prevents melanoma growth^22^. Interestingly, silencing PDK1 enhances anoikis of colorectal cancer cells through the upregulation of reactive oxygen species (ROS)^23^. Therefore, it has been suggested that targeting tumor metabolism is a promising strategy for cancer therapy^24^.

ROS are generated as a result of increased metabolism or cellular stress, and disturbance in ROS homeostasis determines cancer pathogenesis^25^. For example, mitogen-activated protein kinase-regulated ROS production cooperates with antiapoptotic proteins to maintain melanoma cell viability^26^. Klf9 deficiency or treatment with antioxidants inhibits BRAF V600E-induced melanocytic proliferation^27^. Although the presence of ROS is an important etiological factor in melanoma progression, the mechanism by which melanoma cell-derived ROS arise and contribute to metastasis has not been fully described. In this study, we determined that ARNT deficiency has an important impact on mitochondrial function, which in turn controls ROS levels in melanoma. We found that dysregulation of PDK1 resulting from ARNT depletion caused the cells to promote glucose uptake and mitochondrial activation, followed by ROS production and triggered tumor metastasis. The downregulation of ARNT and PDK1, accompanied by ROS production, indicates a high incidence of tumor metastasis.

## Materials and methods

### Cell lines and reagents

The cell line of human melanoma cells A375 (ATCC CRL-1619) and A2058 (ATCC CRL-11147) was grown at 37 °C under 5% CO_2_ in 10 cm plastic dishes containing 10 ml of Dulbecco’s modified Eagle’s medium supplemented with 5% fetal bovine serum, 100 μg/ml streptomycin, and 100 units/ml penicillin.

### Plasmid construction

Full details are available in Supplementary Materials and Methods.

### shRNA clones and Lentivirus infection

Full details are available in Supplementary Materials and Methods.

### Reverse transcription-PCR and mitochondrial DNA quantification

Full details are available in Supplementary Materials and Methods.

### Real-time quantitative PCR

Full details are available in Supplementary Materials and Methods. The specific primers of each target gene were shown in Supplementary Table 1.

### OCR and ECAR assay

Full details are available in Supplementary Materials and Methods.

### shRNA clones and Lentivirus infection

shRNA clones were obtained from the National RNAi Core Facility Platform located at the Institute of Molecular Biology/Genomic Research Center, Academia Sinica. Individual clones should be identified by their unique TRC number (e.g., shARNT: TRCN0000003818 and TRCN0000003819, shLacZ: TRCN0000072223, shPDK1: TRCN0000006261). Lentivirus infection was following standard protocol with minor modify. Briefly, 1 × 10^5^ cells were seeding to 6 cm culture dish overnight. Before lentivirus was infected, the culture medium was changed to the fresh medium containing 8 μg/mL polybrene (Sigma Corporation, Cream Ridge, NJ, USA). Lentivirus was added to the cells at M.O.I. = 3. Following additional incubation of infected cells overnight, infected cells were selected with 1 μg/mL puromycin (Sigma Corporation, Cream Ridge, NJ, USA).

### ROS, mitochondrial superoxide and mitochondrial membrane potential detection

Carboxy-H_2_DCFDA (Invitrogen, Life technologies, Carlsbad, CA, USA) was used to study the intracellular ROS generation. After the 100% confluence was reached, the cells were washed twice with PBS, carboxy-H_2_DCFDA dye was added at a concentration of 20–40 nM, and the plates were kept in a 37 °C, 5% CO_2_ incubator for 30 min. Following incubation, the dye solution was removed; the cells were washed twice with PBS and culture with additional 30 min in fully defined medium. The carboxyl-H_2_DCFDA signal was detected by flow-cytometry (525 nm band-pass filter for FL1). For the mitochondrial superoxide detection, MitoSOX Red mitochondrial superoxide indicator (Invitrogen, Life technologies, Carlsbad, CA, USA) was used to study the mitochondrial superoxide generation. After the 100% confluence was reached, the cells were washed twice with PBS, MitoSOX™ Red mitochondrial superoxide indicator was added at a concentration of 5 μM, and the plates were kept in a 37 °C, 5% CO2 incubator for 10 min. MitoSOX Red, the signal was detected by flow-cytometry (575 nm band-pass filter for FL2). For the mitochondrial membrane potential detection, MitoTracker Red CMXRos (Invitrogen, Life technologies, Carlsbad, CA, USA) dye was used. MitoTracker Red CMXRos was directly added at a concentration of 50–100 nM (50 nM for Flow-cytometry assay; 100 nM for fluorescence microscope observation) for additional 30 min incubation. Mitotracker Red CMXRos, the signal was detected by flow-cytometry (670 nm long-pass filter for FL3). The fluorescence measurement was measured with Beckman Coulter Cell Lab Quanta™ SC flow cytometry (Beckman Coulter, Inc. 250 South Kraemer Boulevard, Brea, CA, USA).

### Western blotting

An analytical 10% sodium dodecyl sulfate poly acrylamide gel electrophoresis (SDS-PAGE) was performed, and 30 μg of protein was analyzed, unless stated otherwise. For immuno-blotting, proteins in the SDS gels were transferred onto a polyvinylidene difluoride membrane by an electroblot apparatus. Antibodies against ARNT (Cell Signaling Technology, Inc., Danvers, MA, USA), α-tubulin (Sigma Corporation, Cream Ridge, NJ, USA), N-cadherin, β-catenin, E-cadherin, vimentin, fibronectin, integrin β1, and phospho-FAK^Y397^ (Epitomics, Inc., Burlingame, CA, USA), NQO1 (Proteintech Group Inc., W Campbell Park, Chicago, USA), Complex I subunit (NDUFB8), Complex II subunit (SDHB), Complex III subunit (UQCRC2), Complex IV subunit II (COX II), and ATP synthase subunit alpha (ATP5A) as an optimized premixed cocktail (Abcam Inc., Cambridge, UK) were used as the primary antibodies. Mouse or rabbit IgG antibodies coupled to horseradish peroxidase were used as secondary antibodies. An enhanced chemiluminescence kit (Pierce, Rockford, IL) was used for detection.

### Transwell migration assay

Cells were trypsinized and 3.5 × 10^4^ cells were added to the Boyden chambers (8 μm pore size; Millipore, Billerica, MA, USA), in 0.5% FBS containing medium, and assay media with 10% FBS was added to the culture plates. After incubation for 15 h, the nonmotile cells at the top of the filter were removed and the motile cells at the bottom of the filter were fixed with 4% paraformaldehyde and stained with a one-tenth dilution of Giemsa (Sigma Corporation, Cream Ridge, NJ, USA). The number of migrating cells in each chamber was counted in five randomly chosen fields under the microscope for three independent experiments.

### Transwell invasion assay

Cells were plated in serum-free medium on the upper Boyden chamber coated with 100 μl of 10% matrigel, with serum-containing medium in the lower chamber. Two days later, cells on the apical side of each insert were scraped off, and invading cells on the basolateral side of the membrane were fixed and stained as the same as transwell migration assay. The number of invading cells was counted in three randomly chosen fields under the microscope for three independent experiments.

### Tumor metastasis assay in an animal model

Tumor metastasis was determined by intravenous (tail vein) injection of cancer cells, which were treated with NAC (20 mM) and CCCP (10 μM) for 24 h into 6 weeks old male severe combined immunodeficient (SCID) mice. For the lung extravasation assay, 1,1’-dioctadecyl-3,3,3’,3’-tetramethylindocarbocyanine perchlorate (DiI) labeled cells (1 × 10^6^) were resuspended in 100 μl of PBS, then injected into the tail vein of mice. Animals were sacrificed up to 48 h after injection with ethical method. The lungs were fixed with 4% paraformaldehyde, 30% sucrose, and finally embedded in FSC 22 (#3801480) (Leica, CA, USA) for cryosectioned (8 μm). Immunohistochemistry (IHC) was then performed to determine the location of vessel with antibody CD31 (ab28364) (Abcam, MA, USA). Quantification was performed by analyzing at least three sections and six fields to determine the number of tumor cells that underwent extravasation. All mice were obtained from the National Cheng Kung University (NCKU) Laboratory Animal Center (Tainan, TWN). The animal study was approved (Approved No. NCKU-IACUC-107-112) by the IACUC of Laboratory Animal Center, Medical College, NCKU.

### Statistical analyses

Statistical analysis was performed with either *t*-tests (for comparison between two groups), one-way ANOVA analysis of variance (Tukey’s or Newman-Keuls’ post-tests, for comparison among multiple experimental groups) using GraphPad Prism 6.0 software (GraphPad Software, San Diego, CA, USA). A Fisher’s exact test and Kendall’s tau (τ)-b correlation analysis were used to examine the relationship between the expression levels of PDK1, ARNT, and NQO1, and various clinicopathologic features. A *P*-value less than 0.05 was considered significant and is denoted by asterisk (*). The *P*-values less than 0.01 and 0.001 are denoted by ** and ***, respectively. n.s.: no significant difference.

## Results

### Depletion of ARNT promotes mitochondrial ROS production

**Fig. 1:**
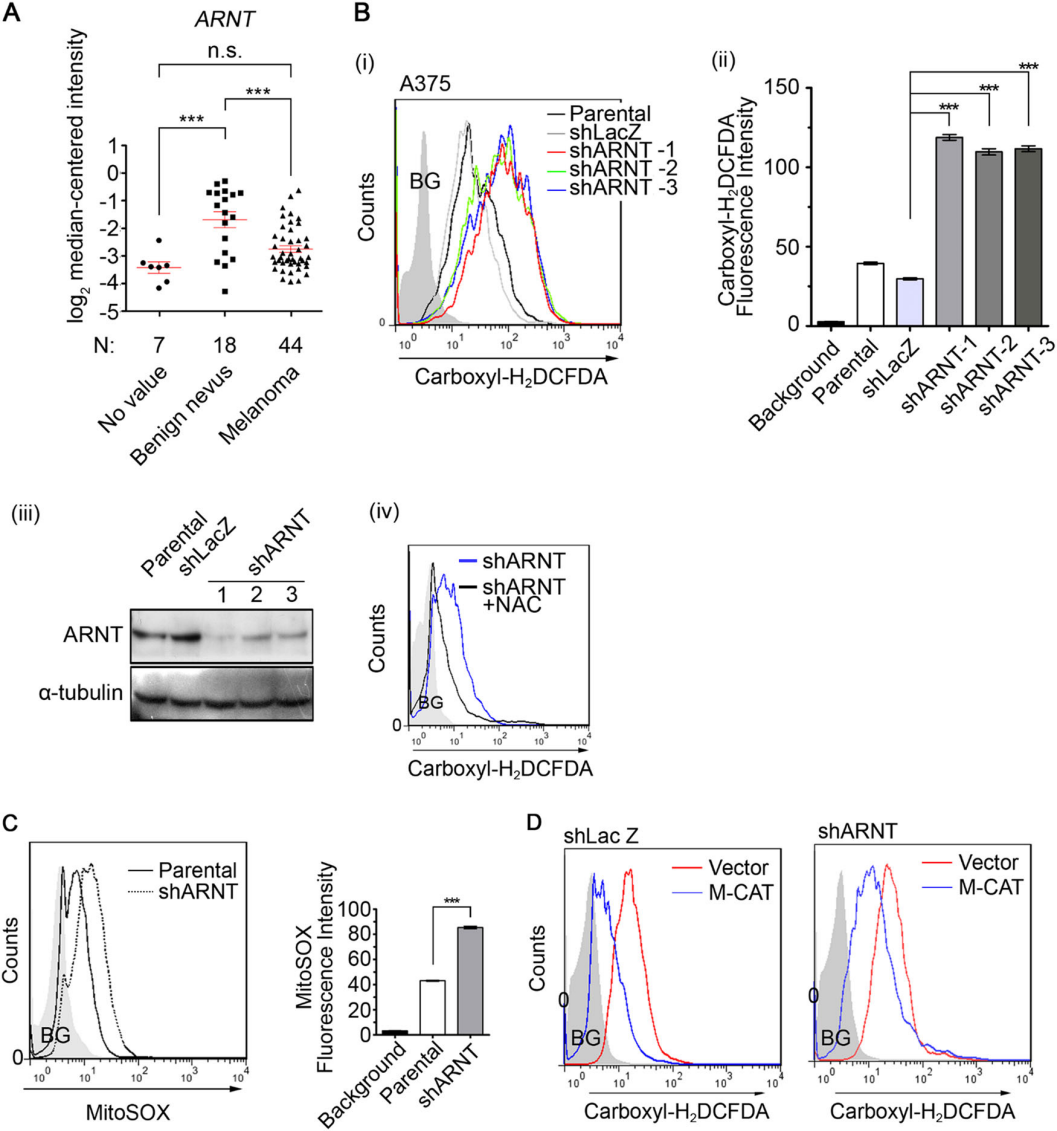
The ARNT deficiency is observed in melanoma tissues and triggers increase of ROS levels. **A** *ARNT* expression was determined from different stages of human melanoma^56^ Values are indicated as the mean ± s.e.m. *P* values were calculated with one-way ANOVA or t-test. Values are indicated as the mean ± s.e.m. ***P < 0.001; n.s.: no significant difference. **B** Carboxyl-H_2_DCFDA staining was performed to quantify the ROS levels in shRNA-mediated ARNT silencing in A375 cells (shARNT). After 30 min incubation, cells were recovered with fully medium. The carboxyl-H_2_DCFDA signal was detected by flow-cytometry (i). The fluorescence intensity of carboxyl-H_2_DCFDA from individual cells was calculated using statistical analysis by Prism 6.0 software (ii). ARNT levels were examined using western blots (iii). Cells were treated with NAC (20 mM)-containing medium for 24 h. The ROS signal was analyzed by flow-cytometry (iv). **C** The mitochondrial superoxide levels were analyzed using MitoSOX Red staining in shARNT A375 cells. After 10 min incubation with 5 μM MitoSOX Red, the signal was detected by flow-cytometry (left panel). The fluorescence intensity of MitoSOX Red from individual cells was quantified using Prism 6.0 software (right panel). **D** Cells were transfected with mitochondrial-targeted catalase expression vector (M-CAT) or empty vector. Following 24 h incubation, the levels of ROS was detected by carboxyl-H_2_DCFDA staining, and analyzed by flow-cytometry.

It is well known that AhR dimerizes with ARNT to trigger melanoma progression, but ARNT also fulfills its multiple functions in tumorigenesis by cooperating with bHLH-PAS family members. To identify the role of ARNT in melanoma progression, the expression levels of ARNT were analyzed in the cancer microarray database from Oncomine 4.0^28^. As shown in Fig. 1A, although the formation of a benign nevus was accompanied by higher expression of *ARNT*, its expression was downregulated in malignant human melanoma. These results were consistent with our previous studies reported that ARNT expression is decreased in late-stage tumors and is correlated with tumor metastasis^19^. In addition, our previous studies revealed that ARNT depletion triggers the ROS production to promote cisplatin-induced melanoma cell death^29^, which suggests that ARNT depletion may trigger excessive ROS in tumor cells. In this study, we further examined the effect of ARNT deficiency on ROS production by using the A375 and A2058 cell lines as models since they are known to harbor the BRAF V600E mutation, which is present in approximately 50% of all melanomas^4^. As shown in Fig. 1B and Supplementary Fig. 1A and B, the knockdown of ARNT using shRNA and siRNA approaches significantly enhanced the ROS levels in melanoma cell lines. The increased amount of ROS in shARNT cells was confirmed by the ROS scavenger N-acetylcysteine (NAC) (Fig. 1B). To investigate the source of ROS induced by ARNT depletion, we examined the production of mitochondrial superoxide using the MitoSOX Red reagent^30^. MitoSOX Red staining demonstrated an increase in mitochondrial superoxide in shARNT cells (Fig. 1C). In addition, the vector containing exogenous mitochondrial-targeted catalase (M-CAT) also repressed the amount of ROS in shARNT cells (Fig. 1D), which suggest that the mitochondrial activation is responsible for ROS generation in ARNT-depleted cells.

### Depletion of ARNT promotes mitochondrial activation

**Fig. 2:**
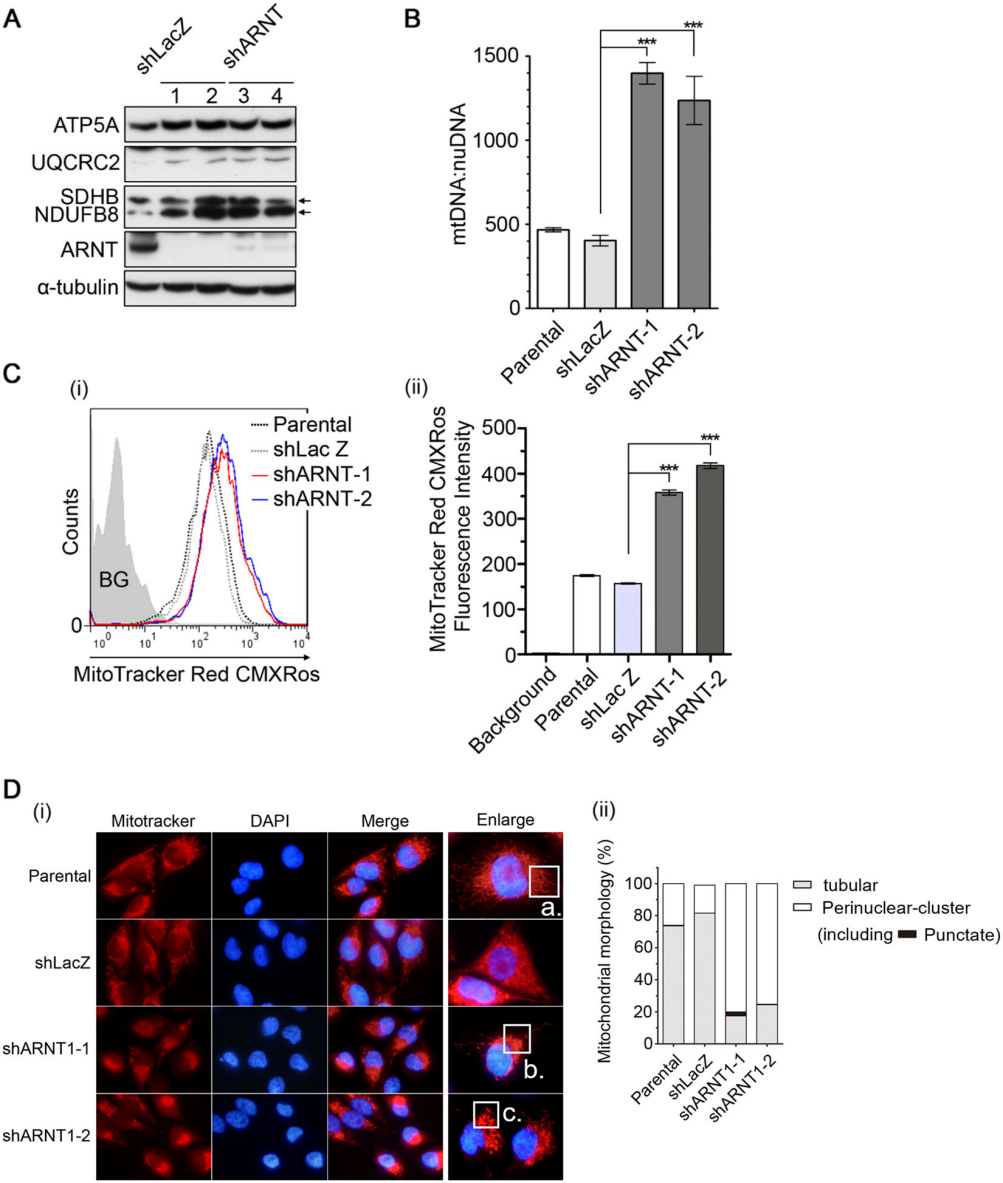
ARNT deficiency induces mitochondrial activity. **A** A375 cell lysates were prepared and subjected to SDS-PAGE and then analyzed by Western blotting with antibodies against ATP5A, UQCRC2, SDHB, NDUFB8, ARNT, and α-tubulin. **B** Mitochondrial DNA (mtDNA) and nuclear DNA (nuDNA) were measured by quantitative PCR from total genomic DNA extracted from cells. The copy number of mitochondrial DNA was normalized by nuclear DNA. **C** Mitochondrial membrane potential was quantified using Mitotracker Red CMXRos staining in shARNT A375 cells. After 30 min incubation with Mitotracker Red CMXRos, cells were harvested and analyzed by flow-cytometry (i). The fluorescence intensity of Mitotracker Red CMXRos from individual cell was statistically analyzed by Prism 6.0 software (ii). **D** Cells were fixed using 4% paraformaldehyde after incubated with Mitotracker Red CMXRos for 30 min. Immunofluorescence images were acquired using a microscope (i). The cell number with tubular, perinuclear and punctate morphology of the mitochondria was counted and statistically analyzed by using more than 100 cells with Prism 6.0 software (ii). a. tubular morphology; b. perinuclear morphology; c. punctate morphology.

To further clarify the mechanism involved in the increase of mitochondrial ROS in shARNT cells, the expression of mitochondrial electron transport chain (ETC) proteins was examined. We found that expression of the ETC proteins ATP5A, UQCRC2 and NDUFB8 was increased in shARNT cells (Fig. 2A). In addition, the mitochondrial DNA copy number was increased in shARNT cells (Fig. 2B), which indicates that the increases in mass and activation of the mitochondria were associated with ARNT deficiency-induced ROS production. Consistently, the mitochondrial membrane potential ΔΨm was also enhanced (Fig. 2C and Supplementary Fig. 2). The previous report suggests that perinuclear clustering of mitochondria was accompanied by the accumulation of ROS^31^. We also found that mitochondria were distributed diffusely throughout the cytosol in parental cells, but increased perinuclear clustering of mitochondria in shARNT cells, which further indicates the generation of nuclear oxidative stress in these cells (Fig. 2D). In summary, these results reveal that increases in mitochondrial mass and activity were responsible for ROS production in ARNT-depleted cells.

### Expression of the antioxidant enzyme NQO1 is inhibited in ARNT-depleted cells

**Fig. 3:**
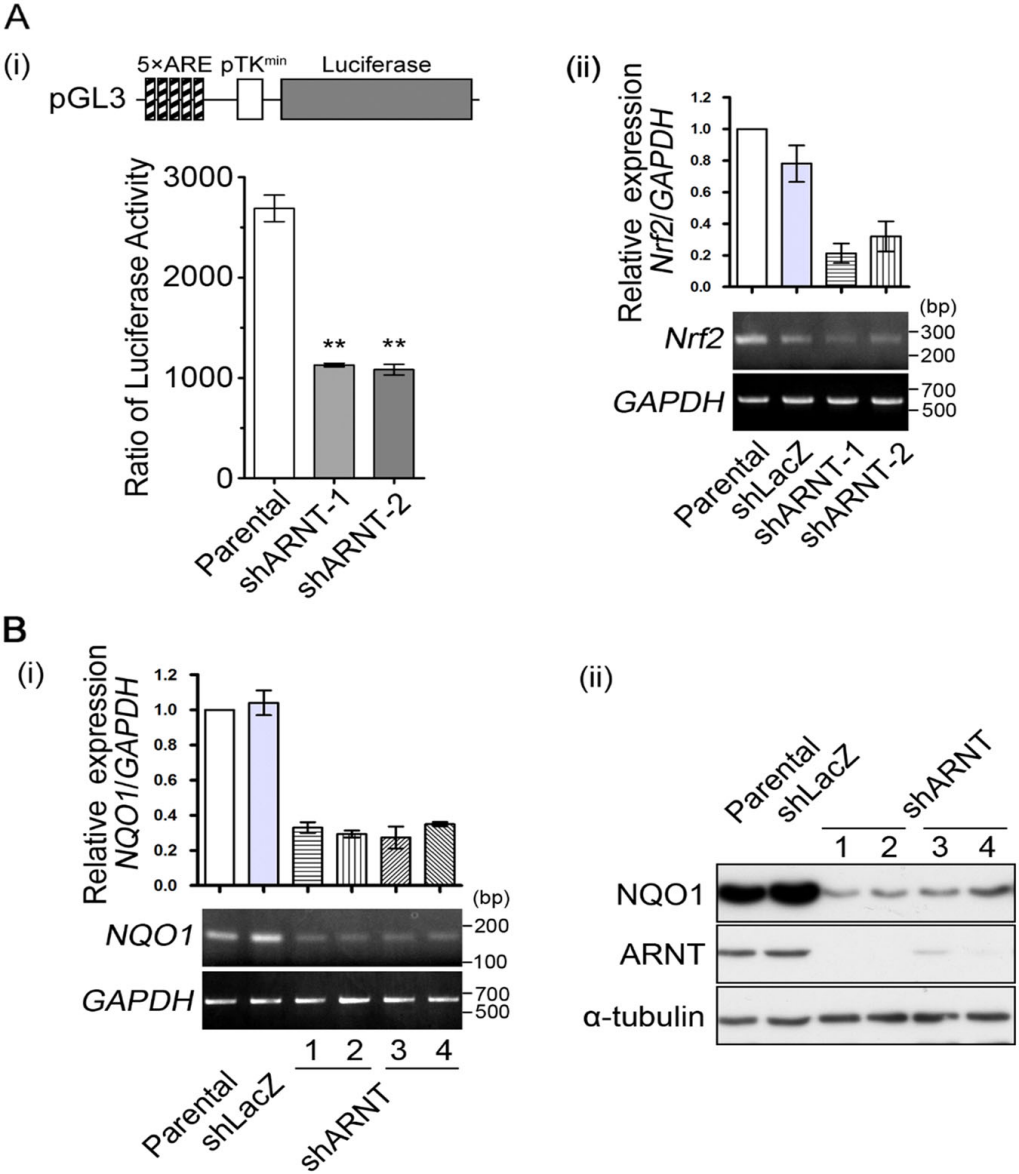
The depletion of ARNT represses NQO1 expression. **A** The construct containing the pTK promoter with 5 repeats of the antioxidant response element (ARE)^57^ and bearing the luciferase gene is presented (i). A375 cells were transfected with 0.5 μg of plasmid by lipofection for overnight. Luciferase activity and protein concentrations were then determined and normalized (i). Values represent the mean ± s.e.m of three determinations. **P < 0.01. Gene expression of *Nrf2* was analyzed in shARNT cells by quantitative real-time PCR (upper panel) and RT-PCR (lower panel). Total RNA was extracted for reverse transcription PCR with *Nrf2* and *glyceraldehyde-3-phosphate dehydrogenase* (*GAPDH*) primers (ii). **B** Gene expression of *NQO1* was analyzed in cells by quantitative real-time PCR (upper panel) and RT-PCR (lower panel) (i). Expressions of NQO1, ARNT and α-tubulin were evaluated by Western blotting with antibodies against NQO1, ARNT and α-tubulin in shARNT A375 cells (ii).

The excessive generation of ROS in cells could be repressed by antioxidant enzymes to reduce the damage to cellular components such as lipids, proteins and DNA^32^. The binding of nuclear factor erythroid 2-related factor 2 (Nrf2) to the antioxidant response element (ARE) within gene promoters regulates the expression of several antioxidative genes such as *NQO1*^33^. To clarify whether ARNT depletion inhibits antioxidant enzymes, which would result in an increase in ROS, we examined the transcriptional activity of Nrf2 using the pTK promoter containing 5 repeated ARE sites. The results showed that the transcriptional activity and gene expression of Nrf2 were reduced in shARNT cells (Fig. 3A), which suggests the possibility that antioxidant genes are expressed at a lower level in ARNT-depleted cells. It is worth noting that ARNT depletion significantly inhibited the expression of NQO1 but not that of genes involved in ROS scavenging such as *SOD1, SOD2*, glutathione-disulfide reductase (*GSR*), and heme oxygenase 1 *(HO1)* (Fig. 3B and Supplementary Fig. 3A). On the other hand, the expression of NOXs including NOX3-5 was significantly downregulated except for NOX1-2 in shARNT cells (Supplementary Fig. 3B). Taken together, these results suggest that the increase in ROS levels in ARNT-depleted cells is at least partially due to the downregulation of NQO1.

### Depletion of ARNT inhibits PDK1 expression and regulates glucose consumption

**Fig. 4:**
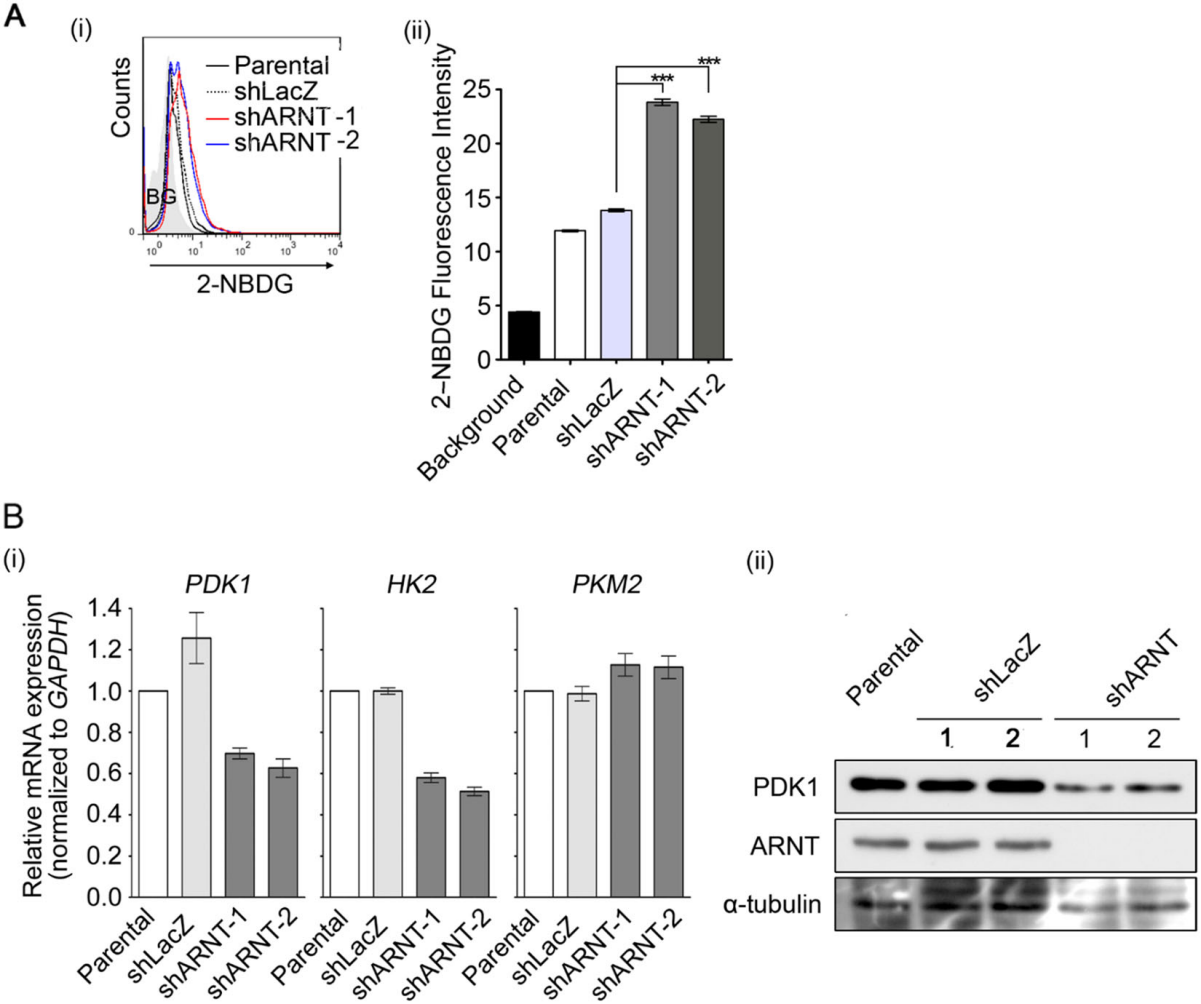
Increase of glucose consumption but downregulation of PDK1 expression is presented in ARNT-depleted cells. **A** The glucose consumption rate was analyzed by 2-NBDG uptake in shARNT cells. A375 cells were incubated in 2-NBDG/PBS (10 μM) solution for 30 min, and then the 2-NBDG signal was analyzed by flow-cytometry (i). The fluorescence intensity of 2-NBDG from 5000 individual cells were statistically analyzed by Prism 6.0 software (ii). **B** Gene expression of *PDK1*, *HK2*, and *PKM2* was analyzed by quantitative real-time PCR in shARNT cells (i). Protein expression level of PDK1, ARNT and α-tubulin was evaluated by Western blotting with antibodies against PDK1, ARNT and α-tubulin in shARNT A375 cells (ii).

The attenuation of mitochondrial function and promotion of glycolytic switch by oncogenic signals have been demonstrated^34^. In addition, our results suggest that the depletion of ARNT improved the mitochondrial function. To investigate whether glucose metabolism is altered in ARNT-deficient cells, the glucose uptake rate was examined using the fluorescent glucose analog 2-NBDG^35^. The glucose consumption assay showed an increase of glucose uptake in shARNT cells (Fig. 4A). Therefore, we further examined the expression of metabolic enzymes that are responsible for glycolysis in shARNT cells. Real-time quantitative PCR revealed the depression of *PDKs* and *HK2* expression in shARNT cells (Fig. 4B and Supplementary Fig. 4). In addition, the decrease in the PDK1 protein level further suggested possible dysregulation of the glycolytic pathway in shARNT cells (Fig. 4B). Indeed, knockdown of ARNT protected cells from glucose and L-glutamine deprivation-induced cell apoptosis (Supplementary Fig. 5), which indicates that ARNT depletion reduces the glucose dependence of these tumor cells. These results reveal that the depletion of ARNT in tumor cells enhances the glucose uptake rate, which reduces glucose dependence.

### Inhibition of mitochondrial activity impairs ARNT depletion-induced cell migration and invasion

**Fig. 5:**
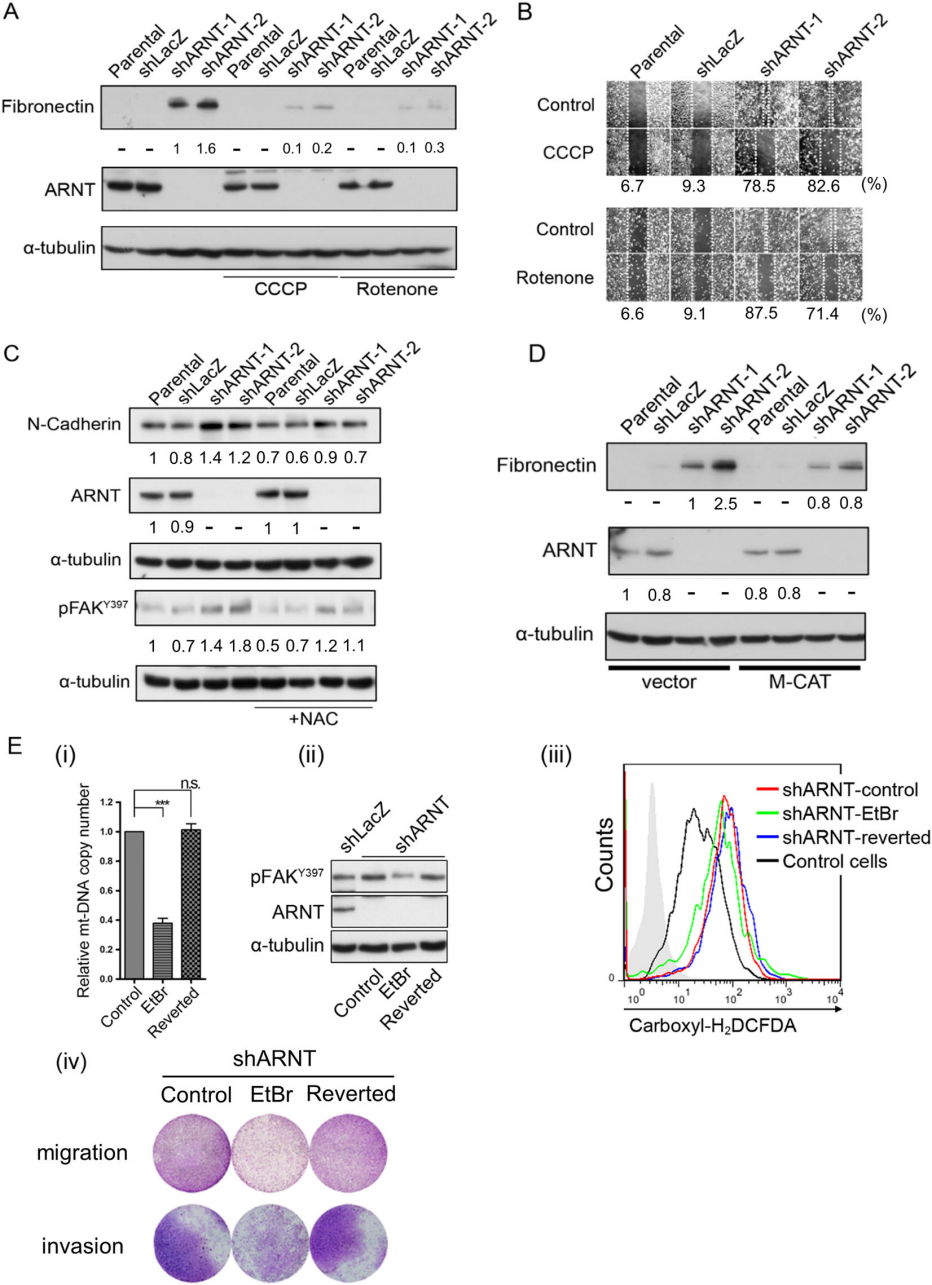
ARNT deficiency-promoted cell migration and invasion are dependent on mitochondrial activity. **A**–**B** A375 cells were treated with CCCP (10 μM) and rotenone (1 μM) for overnight. The cell lysates were prepared and subjected to SDS-PAGE and then analyzed by Western blotting with antibodies against fibronectin, ARNT and α-tubulin. The migration properties were analyzed by wound-healing assays as described in the “Supplementary information”. **C** A375 cells were treated with NAC (20 mM) for overnight. Cell lysates were prepared and subjected to SDS-PAGE and then analyzed by Western blotting with antibodies against phosphorylated FAK^Y397^, N-cadherin, ARNT and α-tubulin. **D** A375 cells were transfected with expression vectors including empty vector and mitochondrial-targeted catalase (M-CAT)^58^. Cell lysates were prepared and subjected to SDS-PAGE and then analyzed by Western blotting with antibodies against fibronectin, ARNT and α-tubulin. **E** ARNT-depleted cells were treated with EtBr (50 ng/ml) for one week. Mitochondrial and nuclear DNA copy number were measured by quantitative real-time PCR from total genomic DNA extracted from indicated cells. The copy number of mtDNA was normalized by nuDNA (i). Cell lysates were prepared and subjected to SDS-PAGE and then analyzed by Western blotting with antibodies against phosphorylated FAK^Y397^, ARNT and α-tubulin (ii). Carboxyl-H_2_DCFDA staining was performed to quantify the ROS levels by flow-cytometry (iii). The migration and invasion properties of EtBr-treated shARNT cells were analyzed by trans-well migration and invasion assays. The migrating and invading assays were described in the “Materials and methods” section (iv). Values are indicated as the mean ± s.e.m. ***P < 0.001; n.s.: no significant difference.

The generation of mitochondrial ROS produced by the respiratory chain during oxidative phosphorylation is associated with cellular glucose uptake^36^. To investigate whether the disruption of mitochondrial oxidative phosphorylation is associated with shARNT-reduced glucose dependence, shARNT cells were treated with inhibitors of oxidative phosphorylation such as carbonyl cyanide m-chlorophenyl hydrazone (CCCP). As shown in Supplementary Fig. 6A, shARNT alleviated cell death upon glucose deprivation, but cell death was restored in CCCP-treated cells. In addition, CCCP also inhibited shARNT-enhanced mitochondrial membrane potential (Supplementary Fig. 6B). These results show that mitochondrial oxidative phosphorylation is essential for ARNT depletion-induced glucose independence. Next, we sought to further clarify whether oxidative phosphorylation also contributes to ARNT depletion-associated tumor metastasis. As shown in Supplemental Fig. 7A and B, the depletion of ARNT significantly enhanced cytoskeletal arrangements and FAK activation, which suggests that ARNT depletion may promote cell mobility. In addition, the depletion of ARNT also induced fibronectin expression and cell migration, but this was reversed by treatment with CCCP and the ETC complex I inhibitor rotenone (Fig. 5A and B). To further clarify whether ARNT deficiency-induced mitochondrial activation was correlated with ROS production followed by cell migration and invasion, the expression levels of EMT markers were examined. The elimination of ROS using NAC or M-CAT overexpression significantly repressed the increases in N-cadherin, pFAK^Y397^ and fibronectin in shARNT cells (Fig. 5C and D). To confirm the correlation between mitochondrial dysregulation and shARNT-induced cell migration and invasion, ethidium bromide (EtBr) was used to induce mitochondrial DNA depletion (Fig. 5E)^37^. As shown in Fig. 5E, EtBr inhibited FAK activation, ROS production, cell migration and invasion. This inhibition was reversed when EtBr was removed from the culture media. These results reveal that mitochondrial activity is associated with the production of ROS in response to ARNT depletion-induced cell migration and invasion.

### Depletion of PDK1 promotes ROS production, mitochondrial activity, cell migration, and invasion

**Fig. 6:**
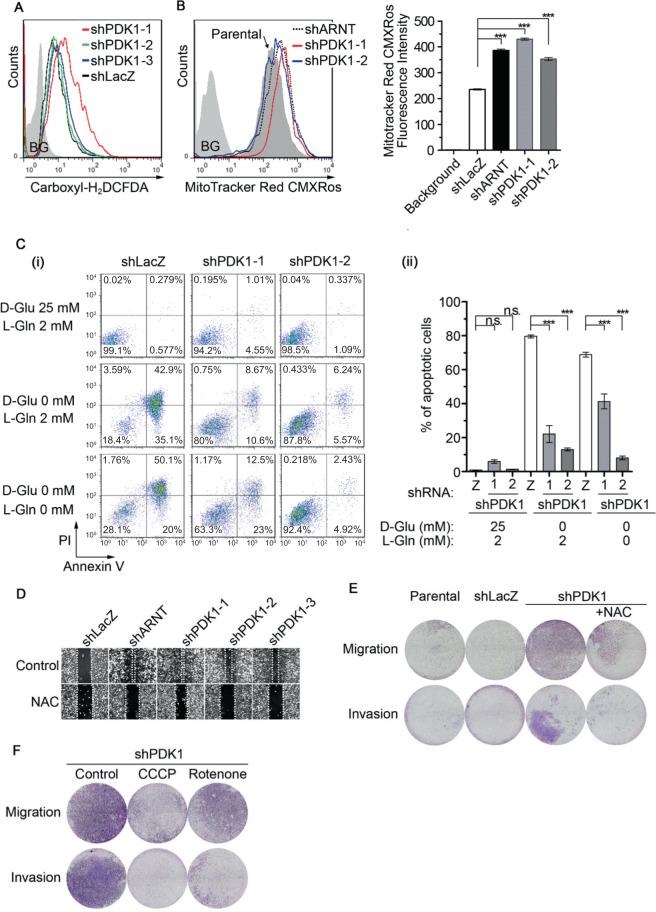
The knockdown of PDK1 enhances ROS levels, glucose independence, and cell migration and invasion. **A**–**B** A375 cells were incubated with Carboxyl-H_2_DCFDA or Mitotracker Red CMXRos for 30 min and then signals were detected by flow-cytometry to evaluate the ROS levels and mitochondrial membrane potential. The fluorescence intensity of Mitotracker Red CMXRos from individual cells was statistically analyzed by Prism 6.0 software (**B**). **C** Cells were cultured in DMEM with the indicated concentration of D-glucose and L-glutamine for 48 h. Total cells were harvested and stained with Annexin V (1:40) and Propidium Iodide (1:1000). The fluorescence intensity of Annexin V and Propidium Iodide were evaluated by flow-cytometry. (i). The percentage of apoptotic cells were statistically analyzed by Prism 6.0 software (ii). **D**–**F** A375 cells were treated with NAC (20 mM), CCCP (10 μM) and rotenone (1 μM) for overnight. The migration and invasion properties of cells were analyzed by wound-healing, trans-well migration and invasion assays as described in the “Materials and methods” section.

The knockdown of ARNT not only inhibited PDK1 expression but also promoted mitochondrial activity (Figs. 2 and 4B). Therefore, we raised the hypothesis that PDK1 may participate in ARNT depletion-activated mitochondrial function. To test this hypothesis, lentiviral shRNA knockdown of PDK1 expression was introduced in melanoma cells (shPDK1) (Supplementary Fig. 8). Consistent with what was observed in shARNT cells, shPDK1 promoted ROS production and mitochondrial membrane potential (Fig. 6A and B). To further confirm the production of ROS was associated with the change of mitochondrial function but not in response to mitochondrial stress, the oxygen consumption rate (OCR) assay was performed. As shown in Supplementary Fig. 9A, both maximal respiration capacity and ATP production were significantly increased in shARNT and shPDK1 cells. On the other hand, extracellular acidification rate (ECAR) assay showed no significant changes in glycolysis and glycolytic capacity in shARNT and shPDK1 cells as compared with shLacZ cells (Supplementary Fig. 9B). These results suggest that the depletion of ARNT and PDK1 impact on the mitochondrial function to promote ROS production. However, the non-obvious glycolytic change in shARNT and shPDK1 cells with downregulation of PDKs that might be manipulated by the increase of glucose uptake (Fig. 4A).

In addition, shPDK1 cells were more resistant to apoptosis than control cells upon glucose deprivation (Fig. 6C), which reveals that depletion of the ARNT/PDK1 axis reduces the dependence of melanoma cells on glucose. The reduction in ROS levels using NAC and the inhibition of mitochondrial activity using CCCP and rotenone, repressed shPDK1-enhanced cell migration and invasion (Fig. 6D–F). Intriguingly, NAC administration improved the translocation of Nrf2 transcription factor from the cytosol into nucleus, suggesting the recovery of NQO1 protein expression in shARNT cells (Supplementary Fig. 10A). In addition, no changes were observed in PDK1 expression in NAC-treated shARNT cells (Supplementary Fig. 10B). These results indicate the reciprocal relationship between ROS and NQO1. On the other hand, PDK1 overexpression had no effect on shARNT-enhanced ROS levels (Supplementary Fig. 10C), suggesting that other PDKs may also contribute to shARNT-triggered ROS production (Supplementary Fig. 4). Taken together, these results demonstrate that depletion of ARNT/PDK1 promotes mitochondrial activity and ROS production, which further suppresses the Nrf2 activation and NQO1 expression to intensify cell migration and invasion.

### Dysregulation of the ARNT/PDK1 axis promotes metastatic extravasation of melanoma through ROS signaling

**Fig. 7:**
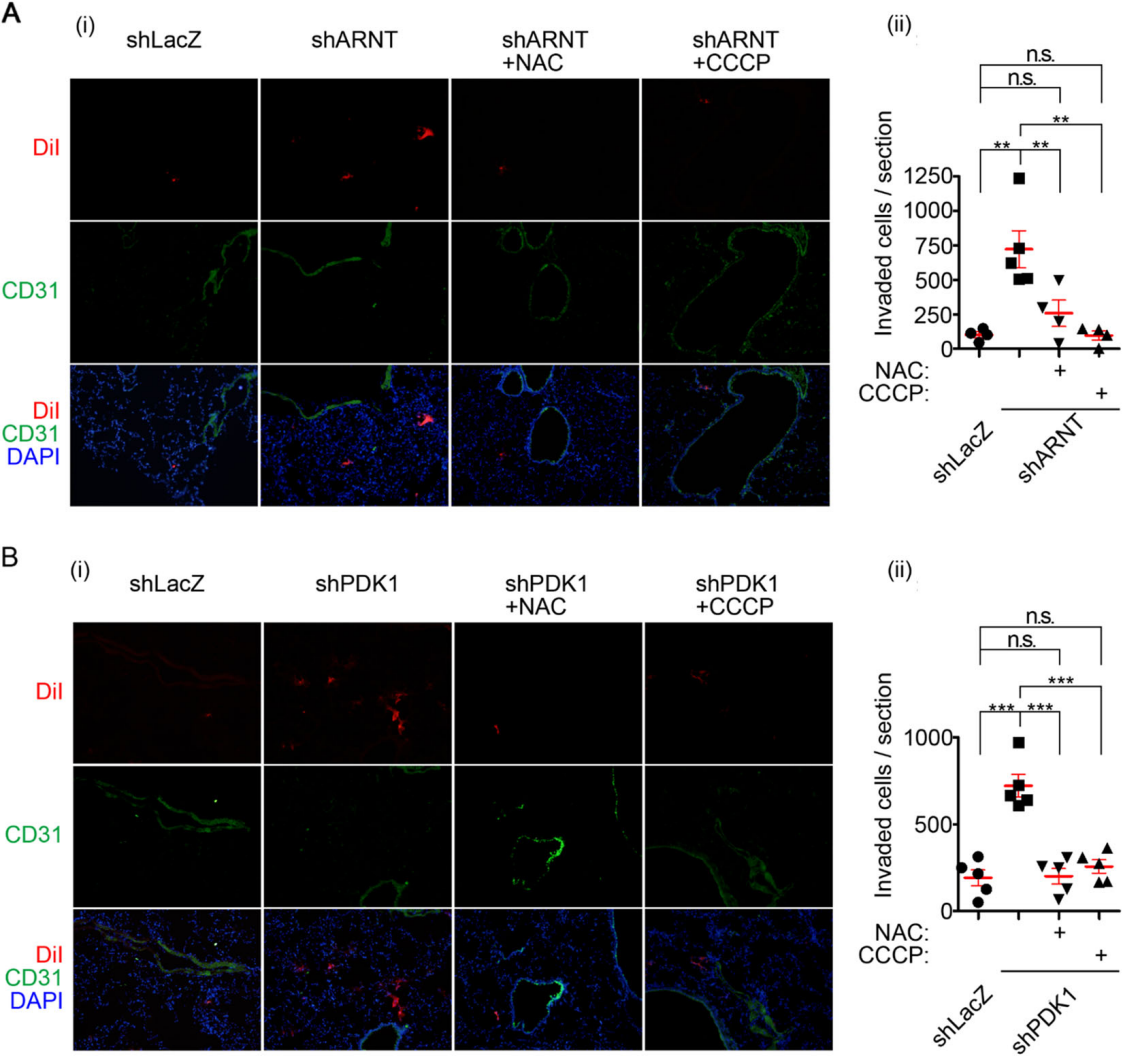
ARNT deficiency-promoted tumor cell extravasation is mediated by oxidative phosphorylation. **A**–**B** Tumor cells penetrate to pulmonary blood vessels that was determined by in vivo extravasation assay. DiI staining of A375 cells were treated with NAC (20 mM) and CCCP (10 μM) for 24 h and then injected intravenously into the tail vein of 6-week-old NOD-SCID mice. At 48 h after injection of tumor cells, the mice were sacrificed for examining of metastatic tumor cells surrounding the lung tissue as described in “Materials and Methods” section. Tumor cell penetration was imaged using a microscope (i). DiI labeled tumor cells (red); CD31 labeled blood vessels (green); DAPI labeled nucleus (blue). The number of tumor cell extravasation was calculated by analyzing at least four sections and six fields (ii); Four or five mice were analyzed for each group.

We previously reported that ARNT expression is decreased in highly invasive and metastatic colorectal cancer^19^. Although downregulation of PDK1 was observed in shARNT cells, the correlation between *PDK1* expression and melanoma progression remains unclear. Next, we analyzed the expression level of *PDK1* using the cancer microarray database from Oncomine 4.0^28^. As shown in Supplementary Fig. 11A, although the formation of benign nevi was accompanied by higher expression of *PDK1*, its expressions was downregulated in malignant human melanoma. In addition, the concurrence of ARNT and PDK1 expressions was observed in melanoma tissues (Supplementary Fig. 11B). On the other hand, the downregulation of *NQO1/NFR2* and their concurrence with *ARNT* were also associated with malignant melanoma (Supplementary Fig. 11C–F). These results show that the downregulation of ARNT/PDK1 expression is accompanied by the risk of tumor invasion and metastasis. We further examined effects of the antioxidant and inhibition of mitochondrial activity on metastatic ability in animal studies using NAC and CCCP, respectively. Although the partial inhibition (<40%) of cell viability in CCCP-treated but not in NAC-treated cells was observed (Supplementary Fig. 12), intriguingly, the in vivo extravasation assay revealed that the tumor cell penetration of blood vessels, which was promoted by shARNT and shPDK1, was significantly inhibited when ROS were scavenged or when mitochondrial activity was inhibited (Fig. 7A and B). These results suggest that ARNT deficiency-regulated pulmonary metastatic extravasation occurs via alterations in mitochondrial activity through PDK1 repression. Taken together, suppression of the ARNT/PDK1 axis promotes melanoma metastasis through increased ROS production.

## Discussion

**Fig. 8:**
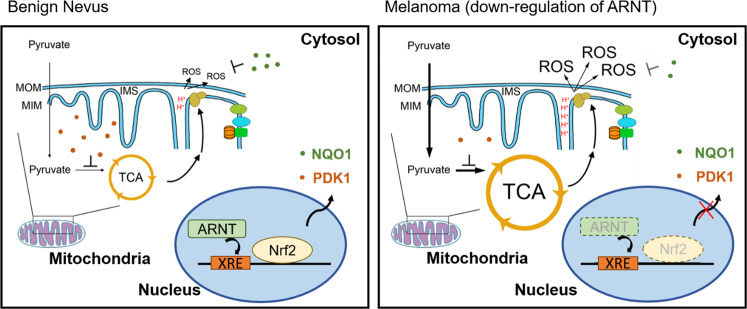
Schematic diagram of the ARNT/PDK1 pathway in regulation of melanoma metastasis. The metastatic properties of tumor cells are promoted in downregulation of ARNT as melanoma occurrence. The ARNT deficiency represses transcription factor Nrf2, resulting in downregulation of PDK1 and NQO1 to promote ROS production and tumor extravasation.

We previously reported that ARNT depletion triggers tumor cell metastasis^19^. We also found that deregulation of ARNT enhances chemotherapeutic drug-induced cancer cell death through increased ROS levels^29^. In this study, the correlation between ARNT deficiency-mediated mitochondrial function and melanoma cell migration, invasion and extravasation triggered by increased ROS levels was further investigated. The results demonstrated that downregulation of the ARNT/PDK1 axis and the increase in ROS may confer tumor cells with the ability to metastasis. Therefore, treatment with antioxidants to prevent chemotherapy-induced melanoma metastasis should be considered in patients using anticancer drugs that target cellular metabolism mediated by the ARNT/PDK1 pathway (Fig. 8).

The BRAF V600E mutation is recognized as the most prevalent mutation, as it is present in approximately half of all patients with advanced melanoma^38^. A recent report indicates that AhR promotes resistance to BRAF-inhibitors in melanoma patients^7^, which suggests that AhR/ARNT confers drug resistance to melanoma cells. Several studies have revealed that, through repression of AhR, the inhibition of AhR/ARNT also stimulates oxidative stress and skin inflammation^39,40^, as well as an increase in ROS production by the loss of ARNT, as shown in our study. However, we further reveal that the dysfunction of ARNT contributed to the metastasis of BRAF V600E-mutated melanoma cells. These results indicate the possibility that targeting AhR/ARNT to overcome melanoma resistance may also be associated with chemotherapy-induced metastasis since ROS levels are increased by AhR/ARNT pathway dysfunction. In addition, the previous report shows that BRAF mutation promotes melanoma tumor growth through reduction of OXPHOS^41^. However, in our study, the depletion of ARNT followed by the reduction of PDK1 in cells with BRAF V600E mutation can further promote oxidative mitochondrial activation. These results indicate that the depletion of ARNT/PDK1 switches OXPHOS activation in BRAF mutation cells to promote melanoma metastasis.

The excess ROS induced by environmental stress, such as chemotherapeutic drugs, cause cell death^17^. However, we found that the increased ROS production in ARNT-depleted cells dramatically induced tumor metastasis, which suggests that the precise amount of ROS is tightly associated with promoting or suppressing tumorigenesis. A recent study showed that increased production of ROS enables and sustains the metastatic phenotype^42^. Therefore, the use of antioxidants to quench ROS has been postulated as a preventive anticancer strategy. Indeed, we found that treatment of tumor cells with NAC, CCCP and rotenone inhibited ARNT or PDK1 deficiency-induced tumor migration and invasion through the reduction of epithelial mesenchymal transition (EMT). These results suggest that tolerance of metastatic tumor cells to ROS allows them to maintain higher ROS levels so that they reach a critical threshold for their viability and metastatic properties.

ROS are produced from peroxisomes, the endoplasmic reticulum, and mitochondria, which is their primary source. In our study, the expression of NQO1, which responds to excessive ROS or xenobiotic stimulation through Nrf2 activation^43,44^, was downregulated by ARNT depletion. Although the antioxidant system has an important role in the development of resistance to chemotherapy and radiation therapy, our results support the possibility that ROS-tolerant tumor cells, such as chemotherapy-surviving cells, may acquire metastatic ability, whereas drug-sensitive cells do not. It is also important to note that antioxidant supplementation fails to benefit patients;^45^ however, several reports have shown that the inhibition of mitochondrial oxidative stress prevents metastasis^46,47^. Thus, the targeting of mitochondrial oxidative enzymes would be a valuable approach to overcome the failure of antioxidants that are unable to target and diminish the ROS generated and localized within mitochondria. For example, targeting the mitochondrial ETC and scavenging mitochondrial superoxide has been used to inhibit metastasis in mice^47^. In addition, we found that PDK1 downregulation, as well as ARNT knockdown caused a switch in glucose consumption to initiate tumor metastasis. These results suggest that those currently conducting clinical trials to test the inhibition of PDK1 for cancer treatment^48^ should be aware of the risk of tumor metastasis. The combination of a PDK1 inhibitor and antioxidants might be a better therapeutic approach for metastatic cancer.

In general, high glucose consumption is a characteristic of tumor cells. We found that ARNT and PDK1 deficiency prevented apoptosis in tumor cells that were deprived of glucose, which indicates that the glycolytic switch from aerobic glycolysis to oxidative phosphorylation is essential for cell survival during metastasis. Our previous studies revealed that ERK activation is responsible for EMT in cells in which ARNT was knocked down.^19^. In addition, the AKT/NF-κB cascade also plays a role in ROS-stimulated EMT^49,50^. These results suggest that the activation of survival pathways by ROS is one of the mechanisms by which ARNT-deficient cells can become resistant to glucose-dependent growth. The switch in the glycolytic pathway in cancers is primarily controlled by the PDK enzymes, which determine the direction of glucose metabolism^51^. In addition to PDK1 in this study, repression of PDK4 also increases mitochondrial respiration and ROS production in mammary epithelial cells^52^. Our studies further revealed that PDK1 downregulation triggered the mitochondrial membrane potential and was associated with an increase in mass and activation of mitochondria in ARNT-knockdown cells. In addition to PDK1, the expression of PDKs was also downregulated in ARNT-depleted cells. Therefore, whether PDKs such as PDK4 also play roles in the regulation of mitochondrial functions to regulate tumor metastasis remains to be investigated. It is worth noting that in clinical databases, PDK1 expression was negatively correlated with melanoma as compared with nevi. Although increased PDK1 has been found in several tumors types^53^, these results highlight the dual functions of metabolic enzymes such as PDK1, as well as ARNT, Sp1 and STMN1, in the promotion of tumor growth and in the inhibition of tumor metastasis^19,54,55^.

In summary, as shown in Fig. 8, ARNT deficiency repressed PDK1 and NQO1 expression in melanoma, which further activated mitochondrial to increase ROS levels; this, in turn, resulted in promotion of tumor metastasis. Therefore, strategies that effectively inhibit ARNT/AhR or eliminate metabolic enzymes by targeting PDK1 might still present the risk of metastatic enhancement. The combination of anti-cancer drugs, including the use of PDK inhibitors and antioxidants, may result in better outcomes after cancer treatment.

## Supplementary information

Supplementary information
