# Supplementary material for: ARNT deficiency represses pyruvate dehydrogenase kinase 1 to trigger ROS production and melanoma metastasis
Source: Oncogenesis. 2021 Jan 14;10(1):11. doi: 10.1038/s41389-020-00299-3 (PMC7809415; doi:10.1038/s41389-020-00299-3)
Supplement: Supplementary file 1 — Supplementary information [file 41389_2020_299_MOESM1_ESM.docx]

**ARNT deficiency represses pyruvate dehydrogenase kinase 1 and triggers ROS production and melanoma metastasis**

Chang et al.

Supplementary Materials and Methods

Supplementary Figures

**Supplementary Materials and Methods**

**Plasmid Construction**

pARE luciferase reporter was constructed by primer assembling using PCR machine and subcloned into BglII and HindIII site of pGL3 according to previous report [1]. Expression vector of including mitochondrial-targeted catalase (M-CAT) was constructed by RT-PCR and subcloned into KpnI and XbaI sites of pcDNA3.1 Myc/His plasmid according to the previous report [2]. All clones were confirmed by DNA sequencing.

**Transfection of cells with siRNA oligonucleotides or plasmids**

Transient transfection of cells with 20 nM siRNA oligonucleotides or plasmids was performed using RNAiMAX or Lipofectamine 2000 (Invitrogen) according to the manufacturer’s instruction with slight modifications. The siRNA IDs were as follows, ARNT (siRNA IDs: #1-HSS100699, #2-HSS100700, #3-HSS100701) (Invitrogen); Negative control siRNAs (siRNA IDs: D-001810-10-50) (Dharmacon, Lafayette, CO, USA). For use in transfection, 3.75 μl of RNAiMAX or Lipofectamine 2000 was incubated with siRNA or plasmids in 1.5 ml of Opti-MEM medium (Invitrogen) for 30 min at room temperature. Following the removal of Opti-MEM medium and replacement with 3 ml of fresh culture medium, the cells were incubated for an additional 24 h, unless stated otherwise.

**Reverse Transcription-PCR and Mitochondrial DNA quantification**

Total RNA was isolated using the TRIzol^®^ RNA isolation reagents (Invitrogen, Life technologies, Carlsbad, CA, USA), and 1 μg of RNA was subjected to reverse transcription-PCR with GoScript™ Reverse Transcription system (Promega Corporation, Madison, Wisconsin, USA). The specific forward and reverse primers were described as the following Supplementary Table 1.

|  | Forward | Reverse |
| --- | --- | --- |
| *PDK1* | CAT GTC ACG CTG GGT AAT GAG G | CTC AAC ACG AGG TCT TGG TGC A |
| *PDK2* | AAGGACACCTACGGCGATG | ATGGAGATGCGGCTGAGG |
| *PDK3* | TTAATAAGTCCGCATGGCGC | TGAAGCATCCCTGGGTTCAC |
| *PDK4* | CAATGGCACAAGGAATCATAG | GGTTCATCAGCATCCGAGTAG |
| *NOX1* | CAA TCT CTC TCC TGG AAT GGC ATC CT | CCT GCT GCT CGG ATA TGA ATG GAG AA |
| *NOX2* | AAG GCT TCA GGT CCA CAG AGG AAA | AGA CTT TGT ATG GAC GGC CCA ACT |
| *NOX3* | ACC GTG GAG GAG GCA ATT AGA CAA | CAG GTT GAA GAA ATG CGC CAC GAT |
| *NOX4* | AGC AGA GCC TCA GCA TCT GTTCTT | TGG TTC TCC TGC TTG GAA CCT TCT |
| *NOX5* | CCT CCT CAT GTT CAT CTG CTC CAG TT | AGG AGG TAG GAC AGG TGA GTC CAA TA |
| *HK2* | GGTGCATCCCCCAGCACTGA | AAAGAGGGGCTGCTGCGTGG |
| *PKM2* | CCATCATGTGGCCCCACCC | AAGGGCCAGGGCCAGAGTCG |
| *NQO1* | CTGGTTTGAGCGAGTGTTC | AATGACATTCATGTCCCCG |
| *HO1* | GTTCATGAGGAACTTTCAGAAG | GGGCAATCTTTTTGAGCAC |
| *GSR* | AGAAGTTTTGATTCAATGATCAGC | AGTCAACATCTGGAATCATGG |
| *SOD1* | GCATCATCAATTTCGAGCAG | ACAGCCTGCTGTATTATCTC |
| *SOD2* | GAACAACAGGCCTTATTCCAC | TGCTACAATAGAGCAGCTTAC |
| *NRF2* | TCATGATGGACTTGGAGC | GGGAGAAATTCACCTGTCTC |
| *mitochondrial chromosom DNA* | AACAAACCTACCCACCCTTAACAGT | TGTGCTATGTACGGTAAATGGCTTT |
| *Nucleus chromosome DNA* | TTCACTTCCCCTTGGCCACAACAT | TGTTCCATGCAGGGGAAAACAAGC |
| *catalase* | GCTGACAGCCGGGATCC | TCCGGACTGCACAAAGGTG |
| *GAPDH* | CCATCACCATCTTCCAGGAG | CCTGCTTCACCACCTTCTTG |

The PCR products were separated by 2% agarose gel electrophoresis and visualized with ethidium bromide staining [3,4,5]

**Real-time quantitative PCR**

Following cDNA synthesis, gene-specific primers were designed using NCBI primer design software. Real-time quantitative PCR was performed in triplicate using a SYBER Green MasterMix (Invitrogen) and an StepOne Real-Time PCR Systems (Applied Biosystems, Carlsbad, CA, USA). Each well contained the following reaction mixture: 1 μl of cDNA, 5 μl of 2 × SensiMix Syber Green PCR reagents (Bioline, London, UK), 4 μl of RNase-free water, 0.5 μl of sense primer, and 0.5 μl of antisense primer. Universal cycling conditions were used. Relative gene expression was calculated using the comparative Ct method. All values were normalized to the housekeeping gene GAPDH.

**OCR and ECAR assay**

The Oxygen consumption rate (OCR) and extracellular acidification rate (ECAR) measurements were performed in a Seahorse XF24 Analyzer (Agilent). 4*104 of cells were seeded in each well of a Seahorse Flux Analyzer plate. Cells were incubated in DMEM without HEPES and sodium bicarbonate for 1 h before oxygen consumption measurement. During the assay, four measurements of the basal level of oxygen consumption were recorded. Subsequently, the complex V inhibitor-oligomycin (5 μM) was injected and mixed, and four measurements were recorded to determine ATP-linked oxygen consumption and proton leak. Next, FCCP (2 μM), a proton uncoupler, was injected and mixed, and another four measurements were recorded to determine maximal respiration capacity. Finally, complex I inhibitor rotenone (2 μM) were injected and mixed, and three measurements were recorded to determine nonmitochondrial oxygen consumption. The ECAR assay was performed in DMEM glucose free medium for 1 h before the assay. During the process, four measurements of non-glycolytic acidification were recorded. Subsequently, the glucose (10 mM) was injected and mixed, and four measurements were recorded to the glycolysis. Next, oligomycin (5 μM) was injected and mixed, and another three measurements were recorded to determine glycolytic capacity. Finally, 2-DG (50 mM) were injected and mixed, and four measurements were recorded to determine glycolytic reverse. Each time point recording was at least quadruplicated. Both the OCR and ECAR output data were analyzed by WAVE software.**Supplementary figures**


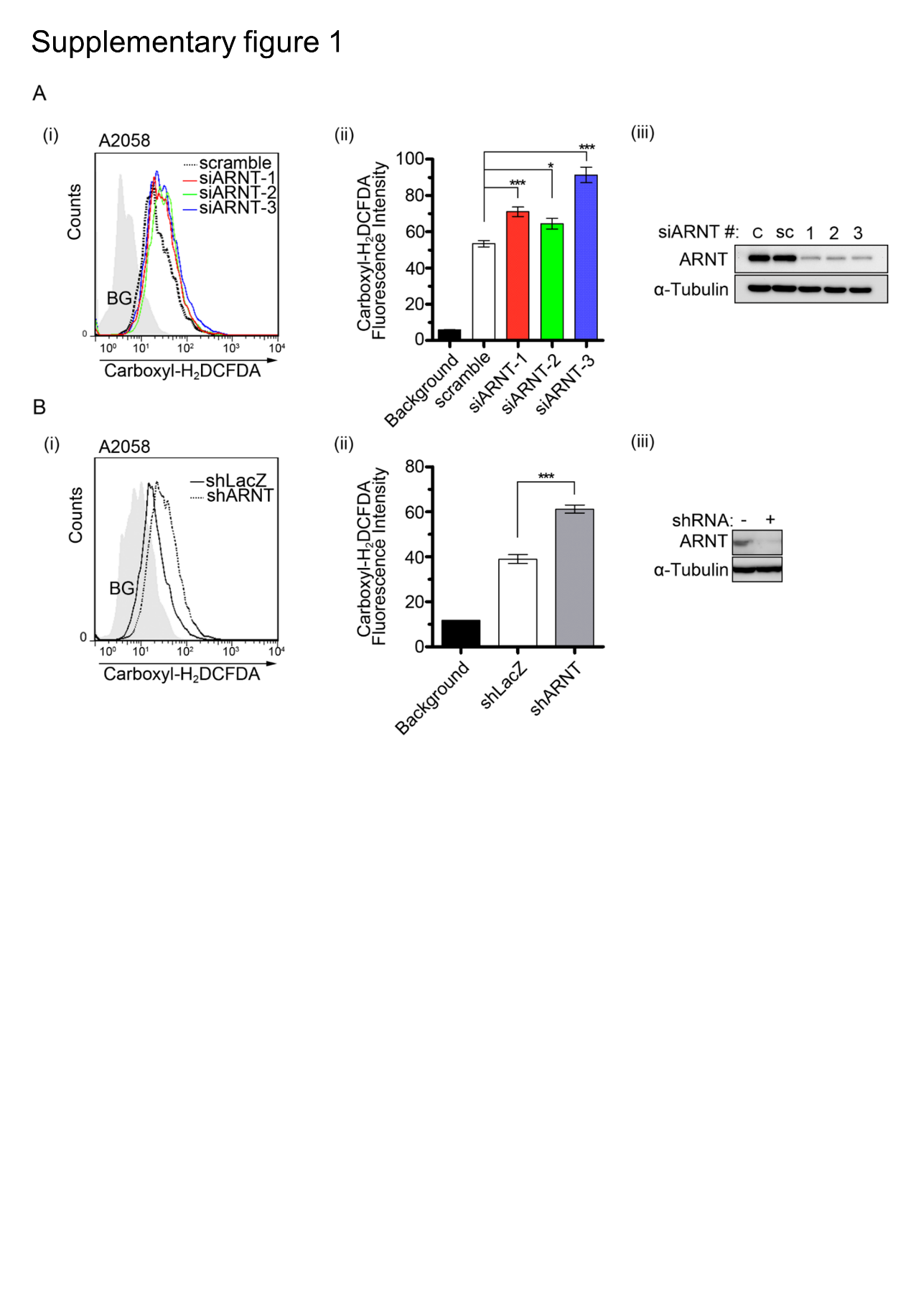


**Supplementary Figure 1.** The knockdown of ARNT promotes ROS generation. (A-B) Carboxyl-H_2_DCFDA staining was performed to quantify the ROS levels in siRNA- (A) and shRNA- (B) mediated ARNT silencing in A2058 cells. After 30 min incubation, the carboxyl-H_2_DCFDA signal was detected by flow-cytometry (i). The fluorescence intensity of carboxyl-H_2_DCFDA from individual cells was calculated using statistical analysis by Prism 4.0 software (ii). Cell lysates were prepared and subjected to SDS-PAGE and then analyzed by Western blotting with antibodies against ARNT and α-tubulin.


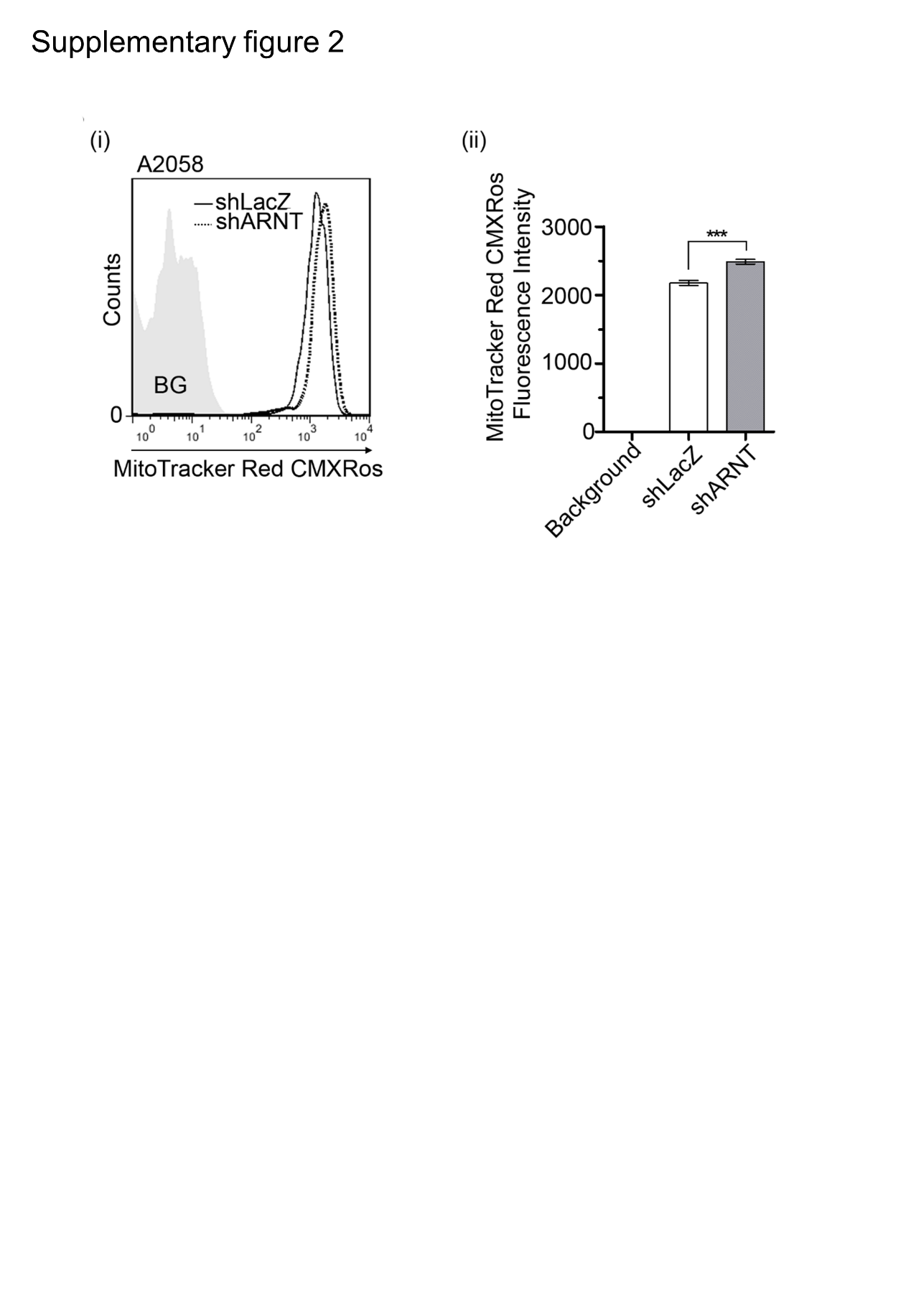


**Supplementary Figure 2.** ARNT deficiency triggers an increase of the mitochondrial membrane potential elevation. (A) Mitochondrial membrane potential was quantified using Mitotracker Red CMXRos staining in shARNT A2058 cells. After 30 min incubation with Mitotracker Red CMXRos, cells were harvested and analyzed by flow-cytometry (i). The fluorescence intensity of Mitotracker Red CMXRos from individual cell was statistically analyzed by Prism 4.0 software (ii).


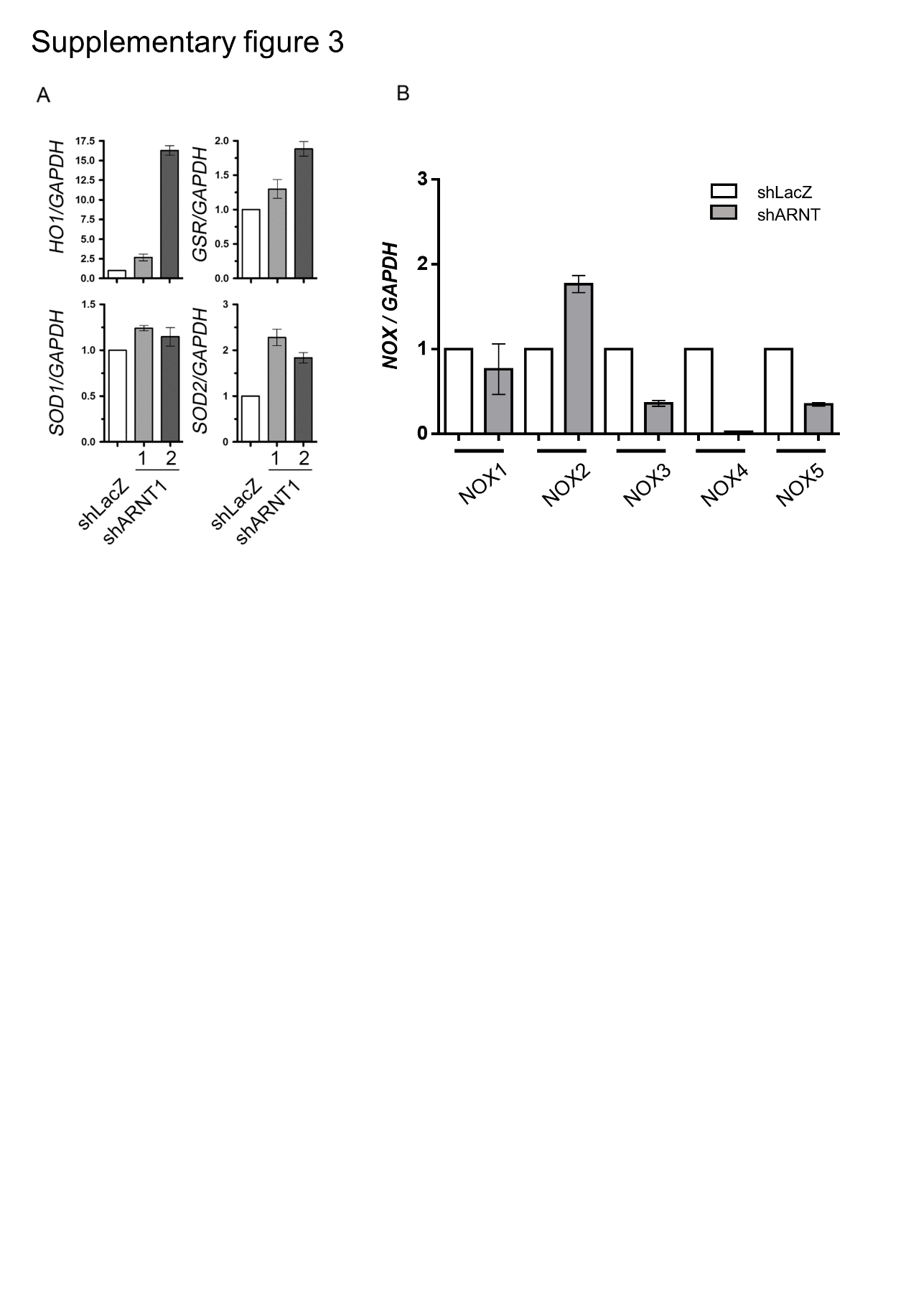


**Supplementary Figure 3.** The mRNA expression of ROS production related genes is examined in ARNT-depleted cells. Total RNA of shLacZ and shARNT were extracted for quantitative real-time PCR (qRT-PCR). (A) Heme oxygenase 1 (*HO1*), glutathione-disulfide reductase (*GSR*), superoxide dismutase 1 (*SOD1*) and superoxide dismutase 2 (*SOD2*) were detected and normalized by glyceraldehyde-3-phosphate dehydrogenase (*GAPDH*). (B) NOXs 1-5 were detected by qRT-PCR and normalized by *GAPDH*.


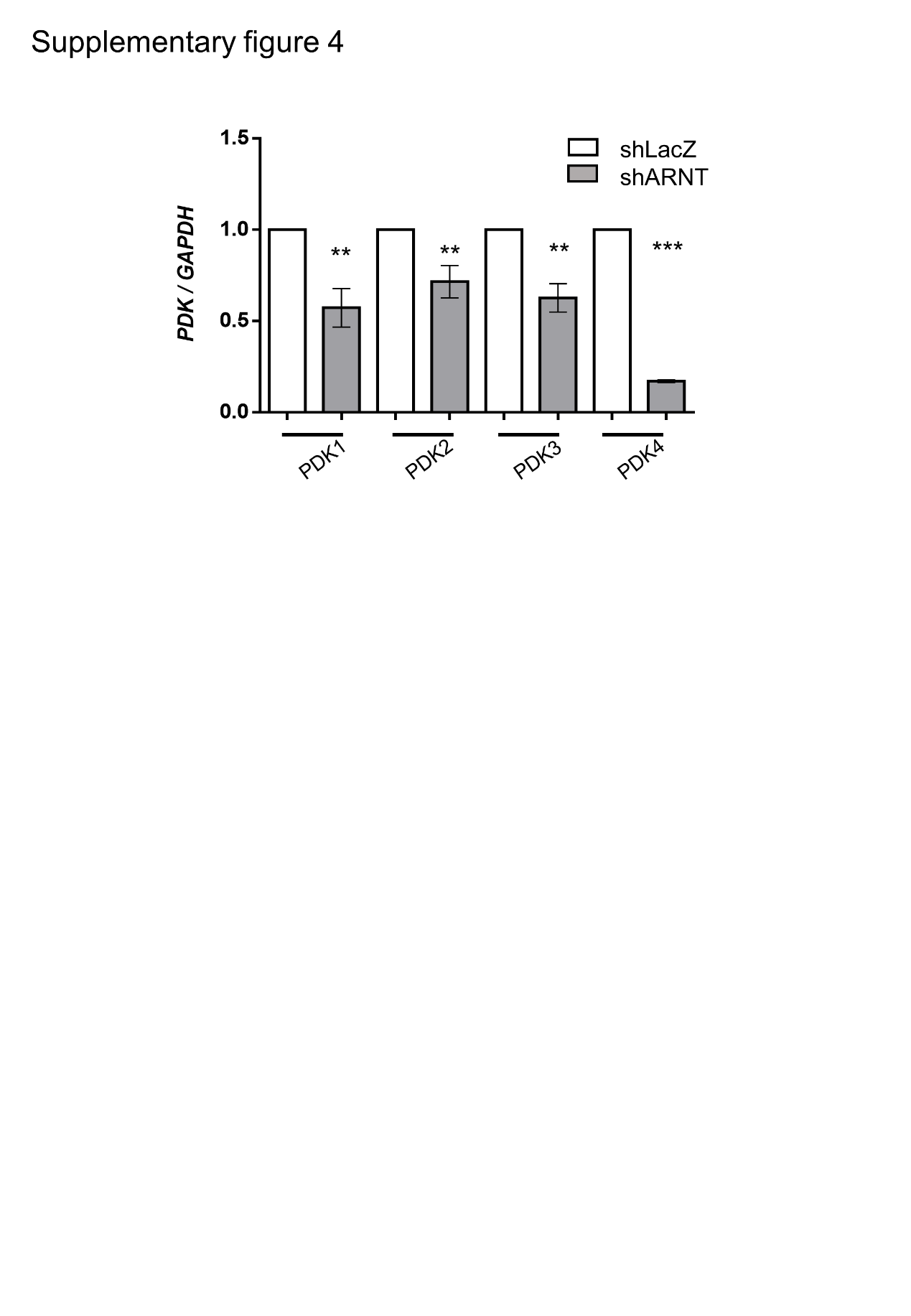


**Supplementary Figure 4.** The mRNA expression of pyruvate dehydrogenase kinases is downregulated in shARNT cells. Total RNA of shLacZ and shARNT cells were extracted for quantitative real-time PCR (qPCR). Data were normalized with GAPDH.


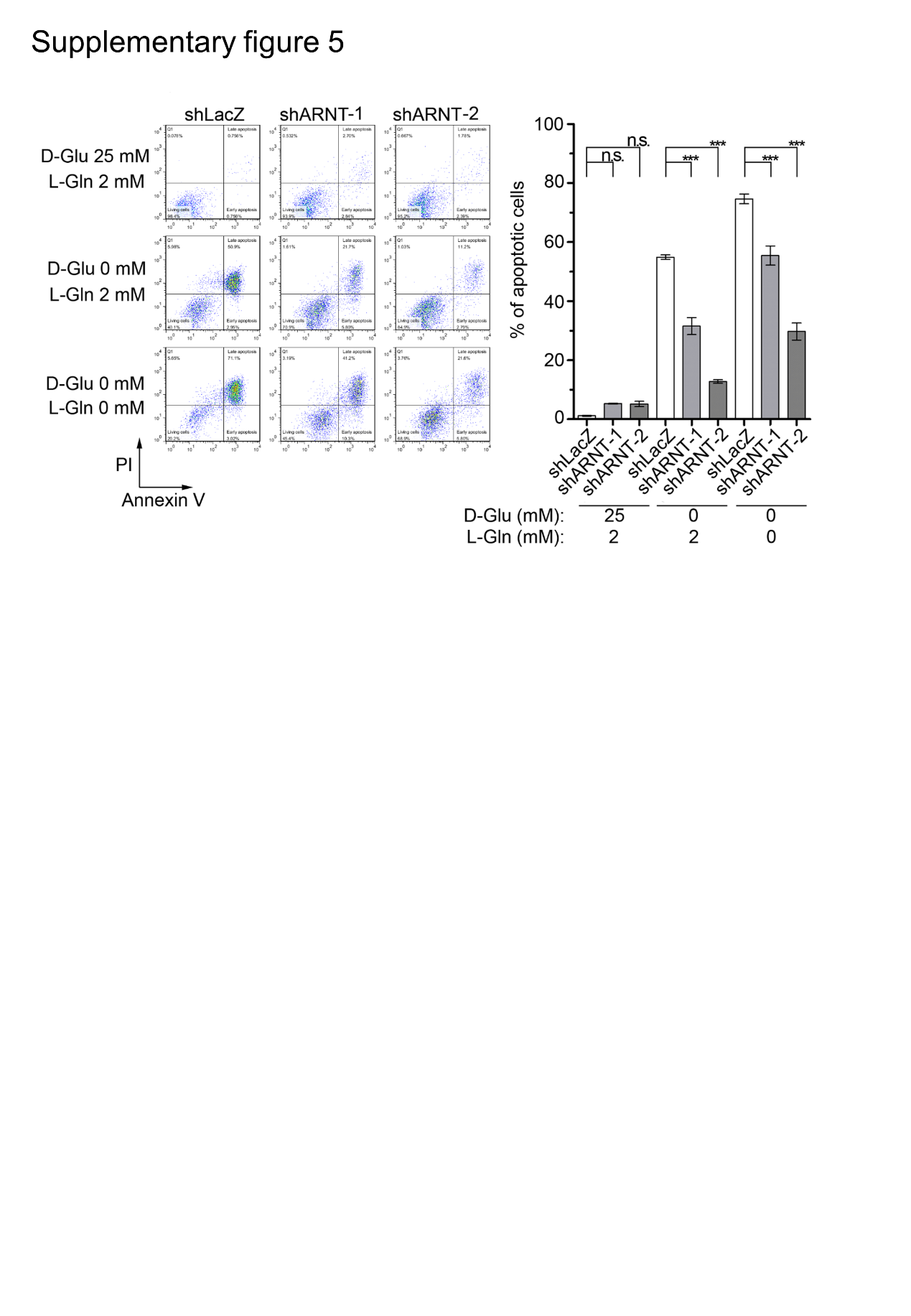


**Supplementary Figure 5.** Glucose deprivation-induced apoptosis is diminished in shARNT cells. A375 cells were cultured in DMEM with the indicated concentration of D-glucose and L-glutamine for 48 h, and then harvested and stained with Annexin V (1:40) and propidium iodide (1:1000). The fluorescence intensity of Annexin V and propidium iodide were evaluated by flow-cytometry (left panel). The percentage of apoptotic cells according to Annexin V-positive population was statistically analyzed by Prism 4.0 software (right panel).


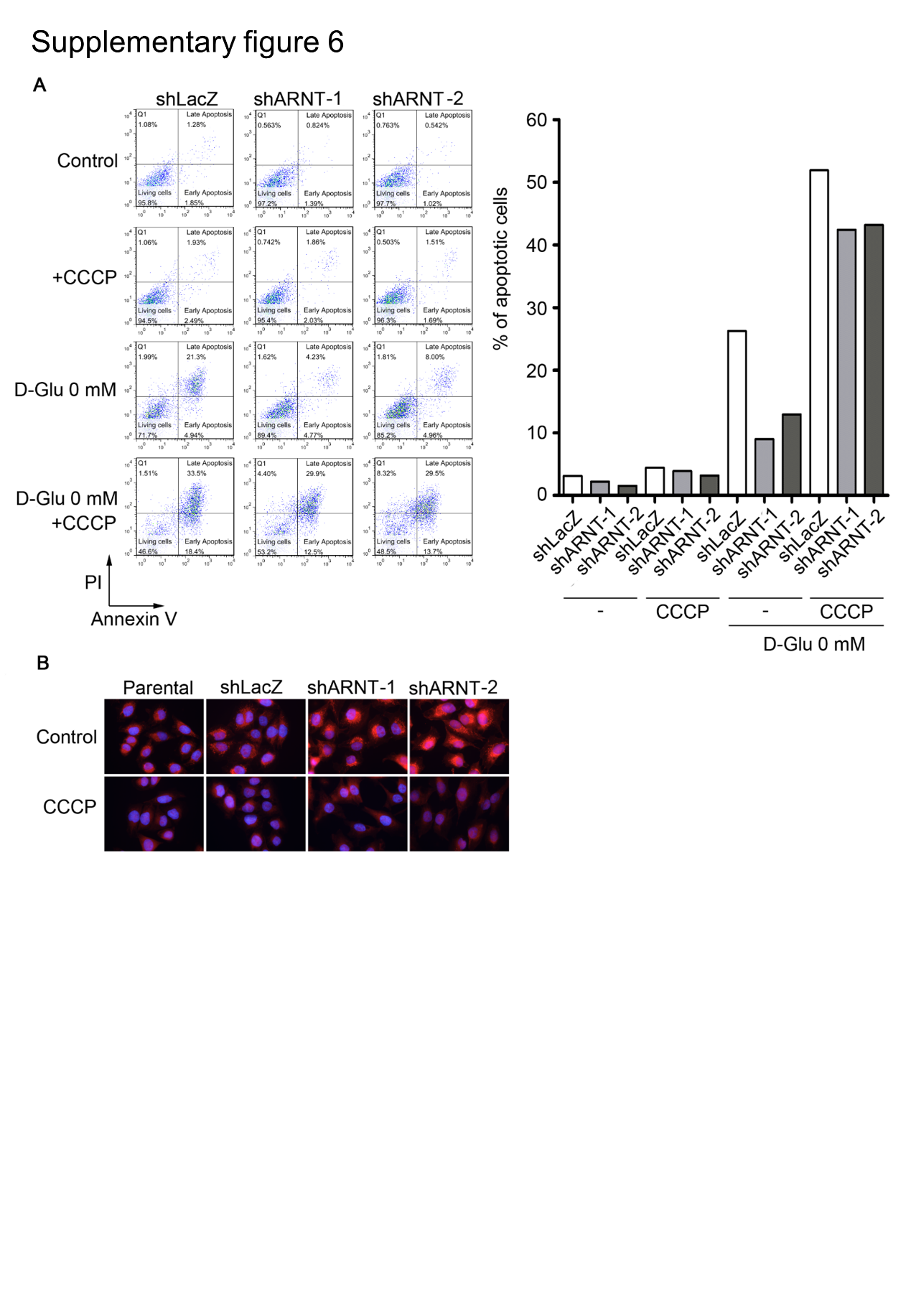


**Supplementary Figure 6.** The maintaining mitochondrial function is essential for anti-apoptosis of shARNT in glucose-deprived cells. (A) A375 cells were cultured in DMEM with or without of D-glucose and CCCP (10 μM) for 24 h. Total cells were harvested and stained with Annexin V (1:40) and propidium iodide (1:1000). The fluorescence intensity of Annexin V and propidium iodide were evaluated by flow-cytometry (left panel). The percentage of apoptotic cells according to Annexin V-positive population was statistically analyzed by Prism 4.0 software (right panel). (B) Cells were treated with CCCP (10 μM), and then Mitotracker Red CMXRos staining was performed to analyze the mitochondrial membrane potential. Immunofluorescence images were acquired using a microscope. Mitotracker Red CMXRos staining (red); DAPI (blue).


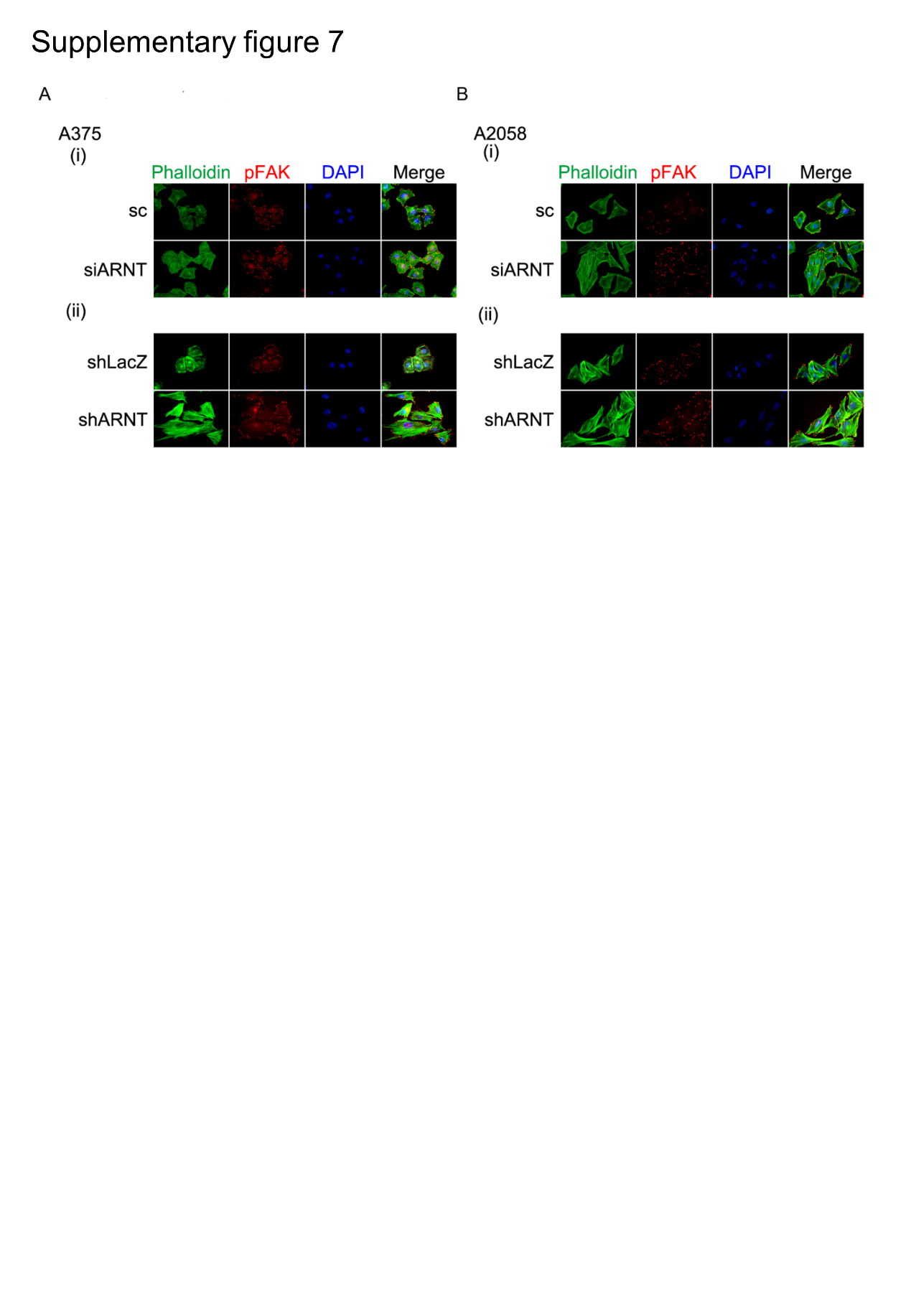


**Supplementary Figure 7.** ARNT deficiency promotes cytoskeleton rearrangement. (A-B) Paraformaldehyde-fixed A375 (A) and A2058 (B) cells were permeabilized and probed with Alexa Fluor^®^ 488-conjugated phalloidin (green) and anti-phosphorylation of FAK^Y397^ (pFAK) antibodies (red). Cells were counterstained with DAPI (blue). Images were taken on a microscope at 600X magnification.


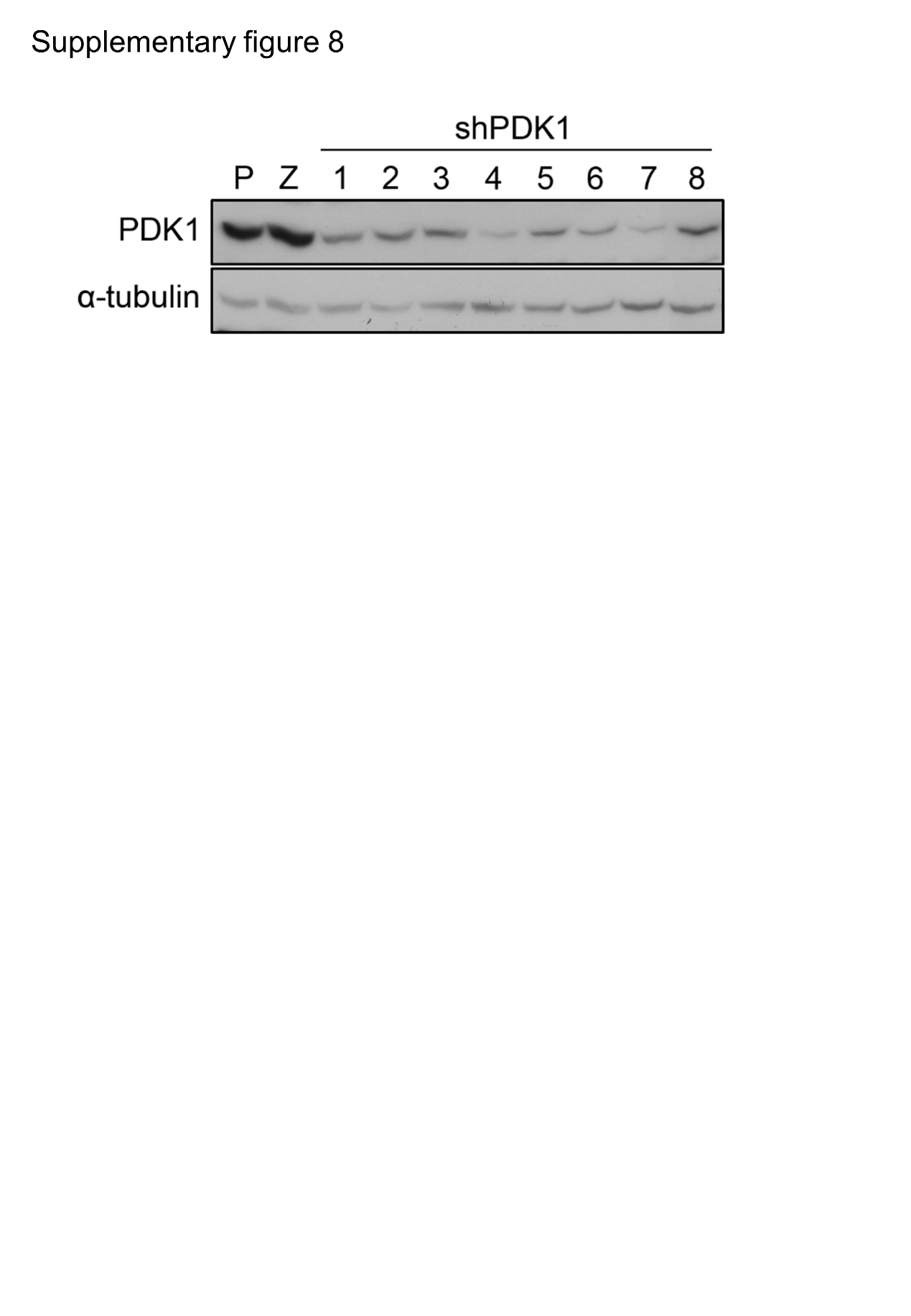


**Supplementary Figure 8.** PDK1 is depleted in A375 cells. Lentivirus-based shRNA against pyruvate dehydrogenase kinase 1 (*PDK1*) was infected into cells. Cell lysates were prepared and subjected to SDS-PAGE and then analyzed by Western blotting with antibodies against PDK1 and α-tubulin.


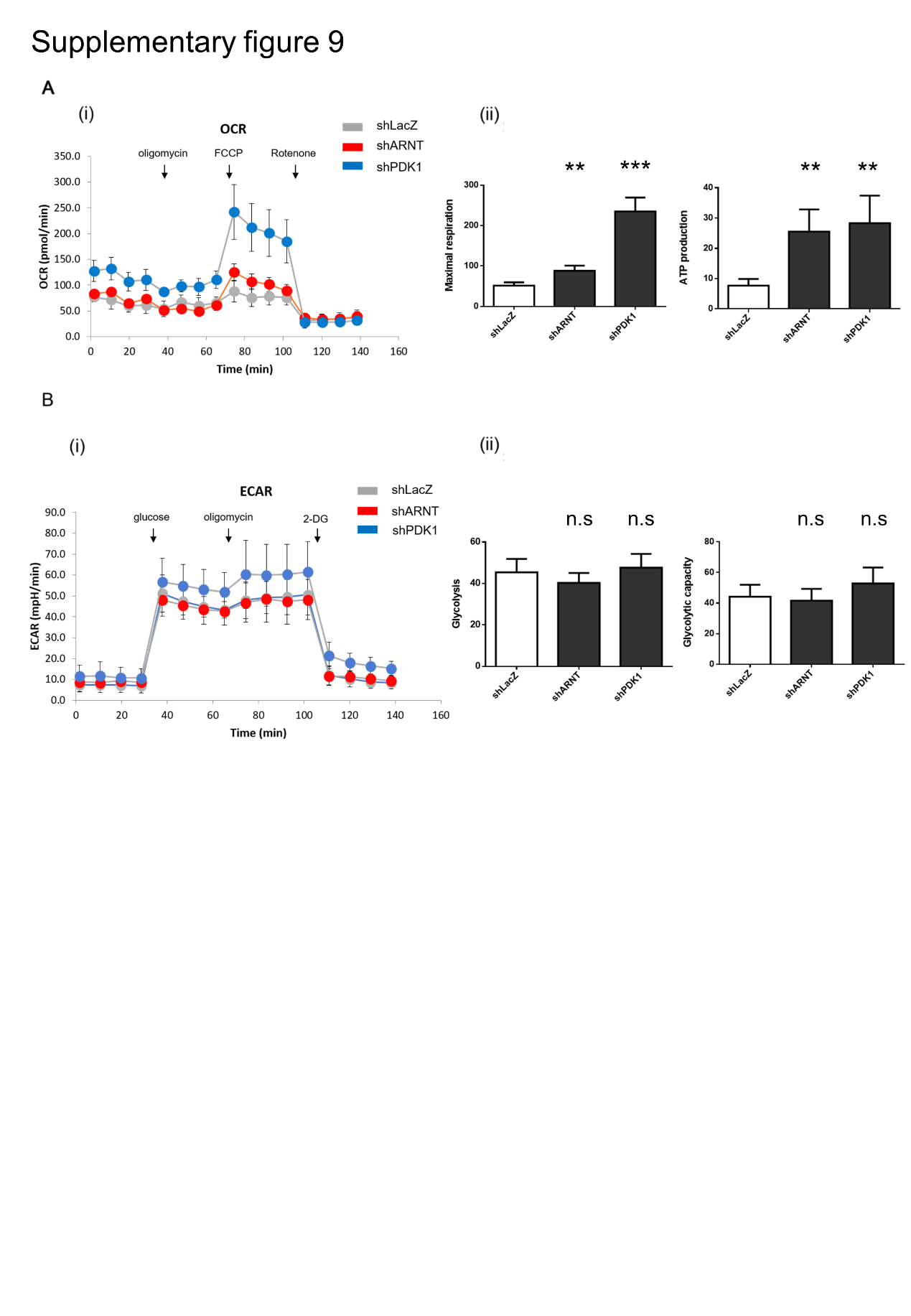


**Supplementary Figure 9. Depletion of ARNT and PDK1 enhances the oxidative phosphorylation but not glycolysis.** (A) Mitochondrial respiration and glycolysis were analyzed using the Seahorse XF analyzer. OCR was measured by treating cells with oligomycin and follow-up treatment with FCCP and ended up with rotenone (i). The maximal respiration and ATP production were measured by WAVE software (ii). (B) ECAR assay were performed in seahorse specific plate, by treating cells with glucose and follow-up treatment with oligomycin and ended up with 2-DG (i). The glycolysis and glycolytic capacity were measured by WAVE software. Each stage was recorded for four times. The assay was performed in quadruplicate.


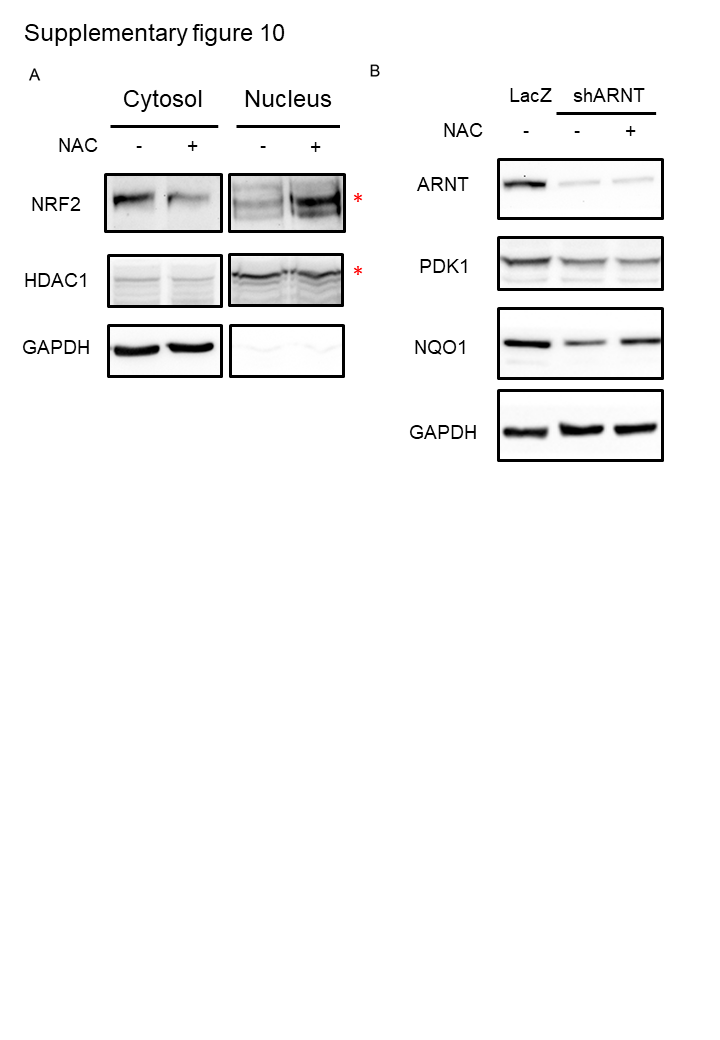


C


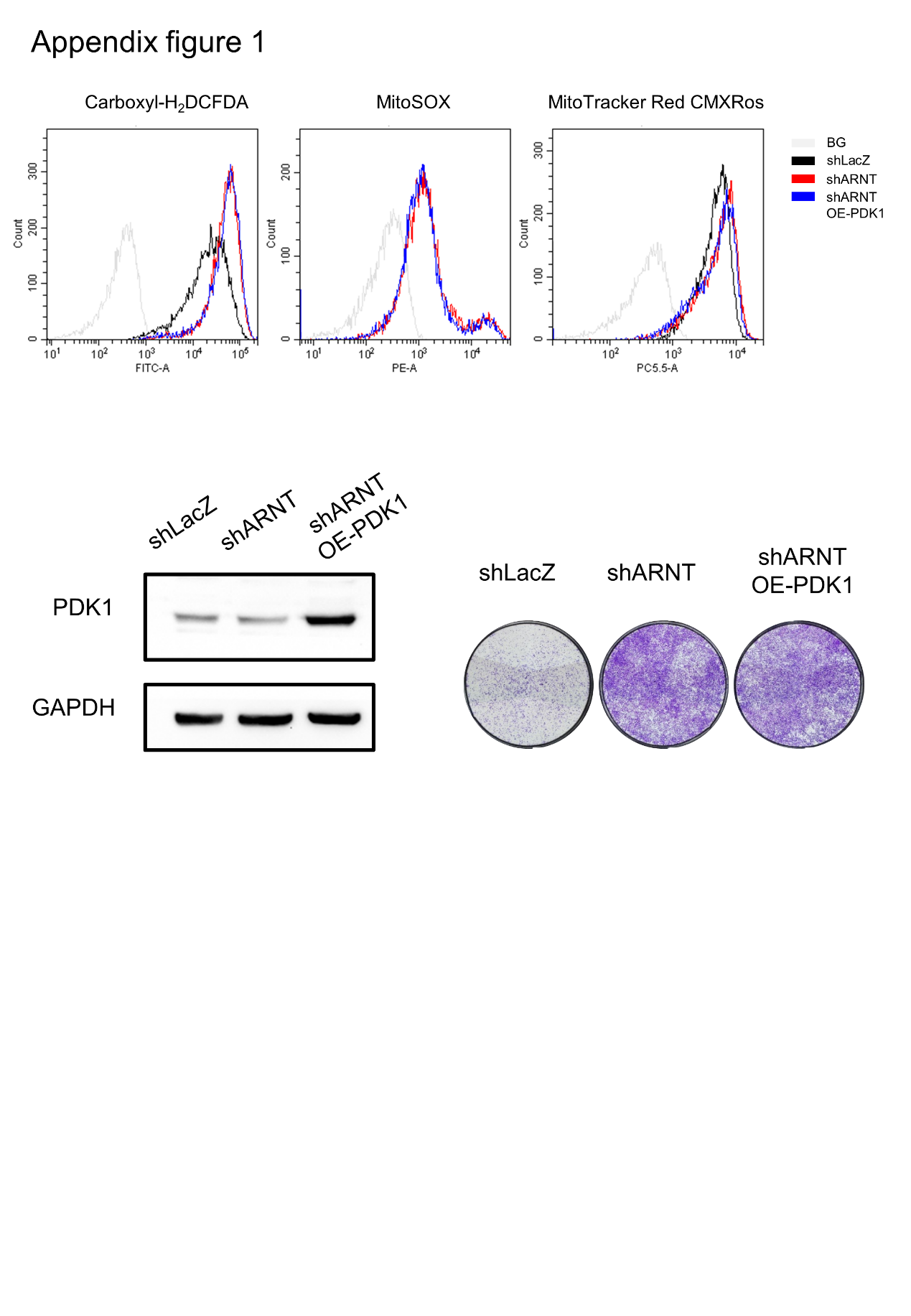


**Supplementary Figure 10. The expression of NQO1 and nuclear Nrf2 is increased in NAC-treated shARNT cells.** (A) Cells were treated with NAC (20 mM) for overnight, and then cytosol and nuclear fractions of proteins were harvested as the following protocol [6]. The expression of Nrf2, HDAC1, and GADPH was examined using western blotting. HDAC1 and GAPDH represented the internal control of nuclear and cytosol fractions, respectively. (B) Total cell lysates were harvested and western blotting was performed by using antibodies against ARNT, PDK1, NQO1, and GAPDH. (C) The PDK1 expression vector was transfected into cells using lipofection method. The overall ROS, mitochondrial ROS and mitochondrial membrane potential were labeled by indicated dye. After incubated for 30 minutes, the fluorescence intensity was detected by flow-cytometry. (OE: overexpression)

**
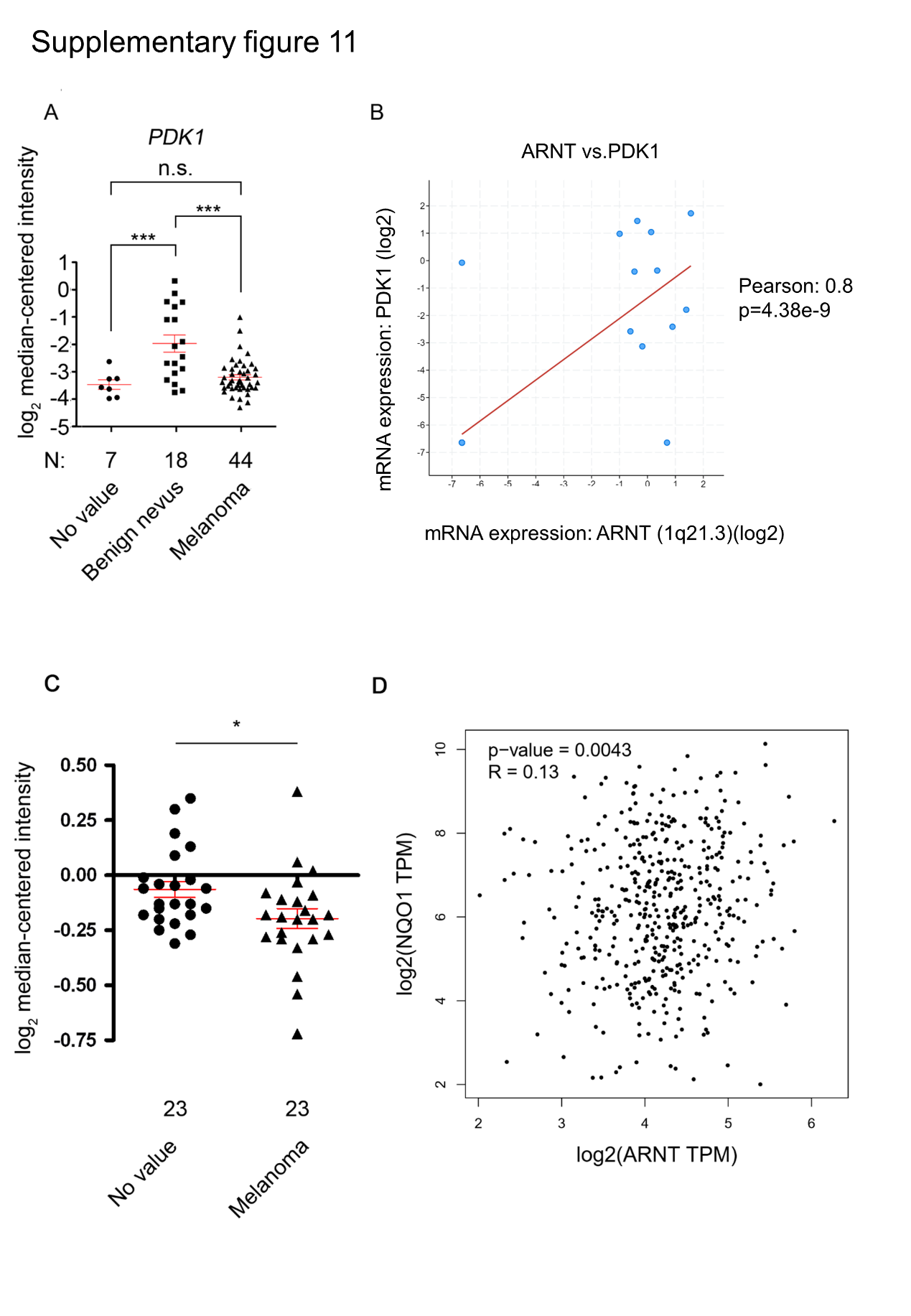
**

**
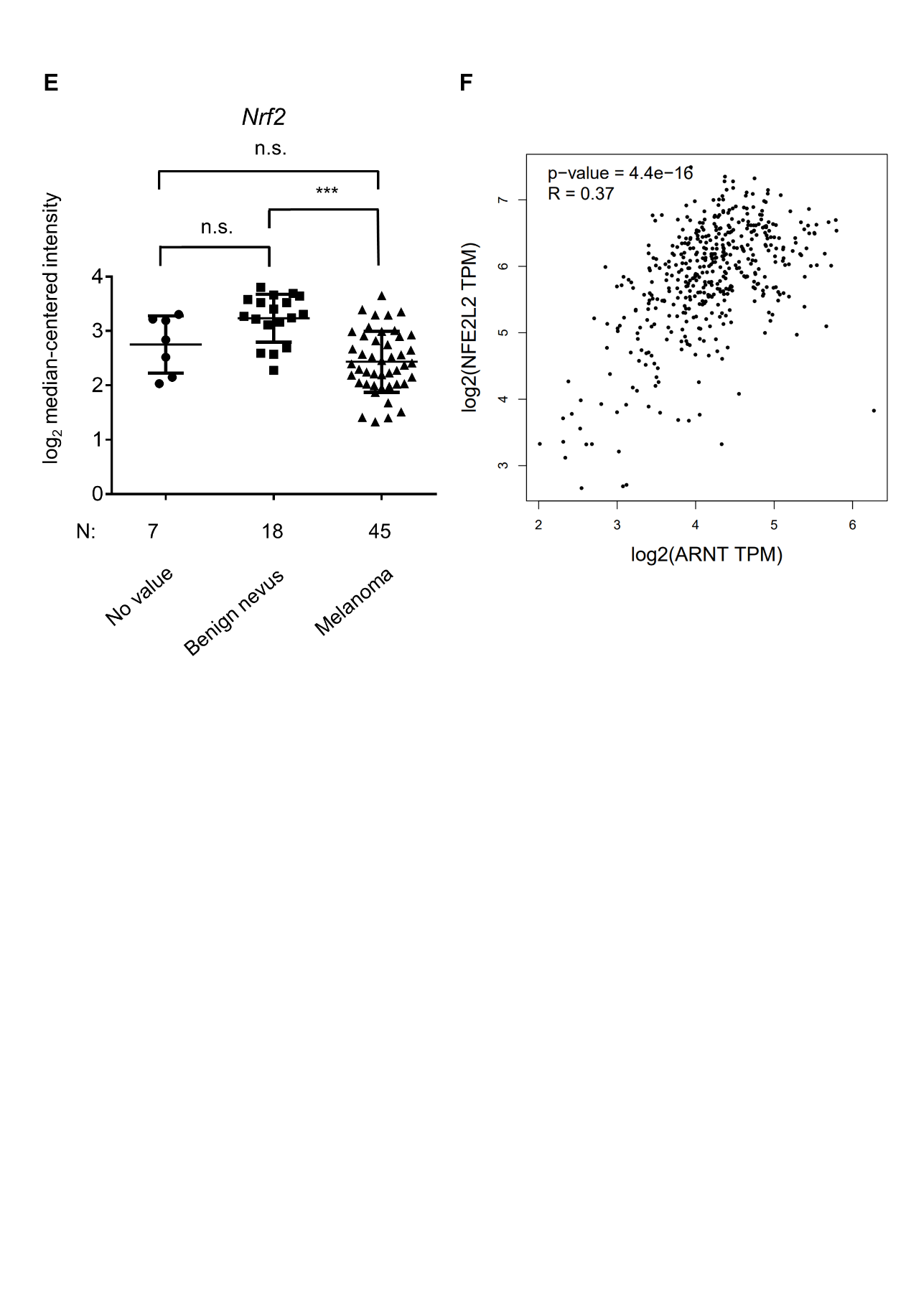
**

**Supplementary Figure 11.** The expression of PDK1 and NQO1 is presented in melanoma tissues. (A, C and E) *PDK1*, *NQO1* and *Nrf2* expression was determined from the database of human benign nevus and melanoma [7]. (B) Dataset from cBioportal (n=13) were included to determine the correlation of ARNT and PDK1. (Pearson’s correlation coefficient (R^2^) is shown in the figure) [8-10]. (D and F) Concurrent expression of ARNT and NQO1/Nrf2 in tumor tissues of melanoma patients (n=597) in TCGA database was quantitated (Pearson’s correlation coefficient (R) is shown in the figures). FPKM: Fragments Per Kilobase of transcript per Million. Values are indicated as the mean + s.e.m. *: *P*<0.05; ***: *P*<0.001.


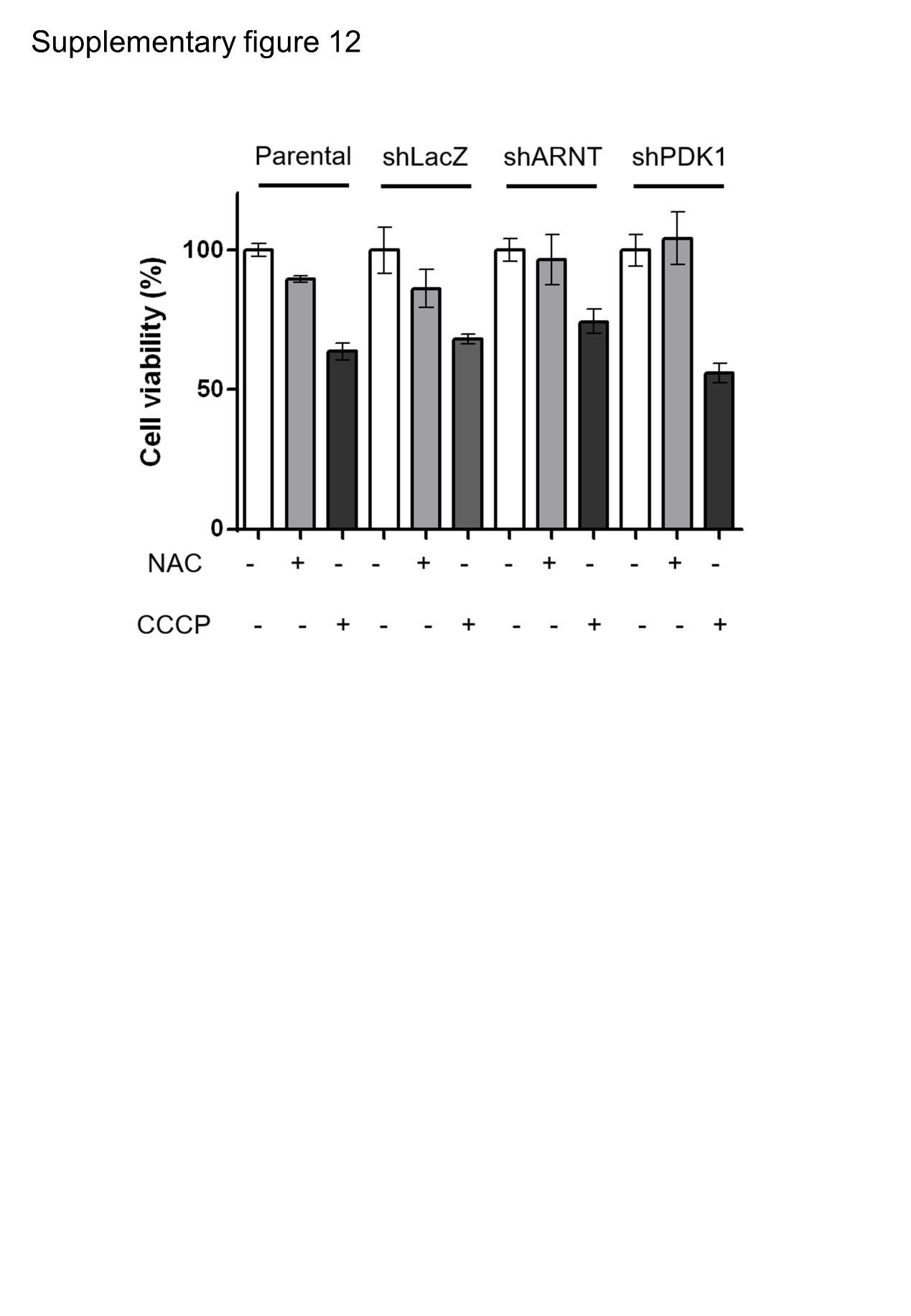


**Supplementary Figure 12. The effect of NAC and CCCP on cell viability is determined using shARNT and shPDK1 cells.** MTT assay was performed following by GOLDBIO protocol to detect the cell viability [11]. 1*10^4^ of parental, shLacZ, shARNT or shPDK1 cells were seeded in 96-wells. After 24 h, cells were then treated with NAC (20 mM) or CCCP (10 μM) for overnight. The crystal was dissolved in DMSO and measured by ELISA reader for the wavelength of 595nm.

**Reference**

1 Simmons SO, Fan CY, Yeoman K, Wakefield J, Ramabhadran R. NRF2 Oxidative Stress Induced by Heavy Metals is Cell Type Dependent. Curr Chem Genomics. 2011; 5: 1-12 .

2 Schriner SE, Linford NJ, Martin GM, Treuting P, Ogburn CE, et al. Extension of murine life span by overexpression of catalase targeted to mitochondria. Science. 2005; 308: 1909-1911.

3 Anderson S. et al. Sequence and organization of the human mitochondrial genome. Nature. 1981; 290: 457-465.

4 Moiseeva O, Bourdeau V, Roux A, Deschenes-Simard X, Ferbeyre G. Mitochondrial dysfunction contributes to oncogene-induced senescence. Mol Cell Biol. 2009; 29: 4495-4507.

5 Zhang J. et al. NOX4-Dependent Hydrogen Peroxide Overproduction in Human Atrial Fibrillation and HL-1 Atrial Cells: Relationship to Hypertension. Front Physiol. 2012; 3: 140.

6 Dimauro I, Pearson T, Caporossi D, Jackson MJ. A simple protocol for the subcellular fractionation of skeletal muscle cells and tissue. BMC Res Notes. 2012; 5: 513.

7 Critchley-Thorne RJ, Yan N, Nacu S, Weber J, Holmes SP, Lee PP. Down-regulation of the interferon signaling pathway in T lymphocytes from patients with metastatic melanoma. PLoS Med. 2007; 4(5): e176.

8 Liang WS. et al. Integrated genomic analyses reveal frequent TERT aberrations in acral melanoma. Genome Res. 2017; 27(4): 524-532.

9 Cerami E. et al. The cBio cancer genomics portal: an open platform for exploring multidimensional cancer genomics data. Cancer Discov. 2012;2(5): 401-404.

10 Gao J. et al. Integrative analysis of complex cancer genomics and clinical profiles using the cBioPortal. Sci Signal. 2013; 6(269): pl1.

11 Song L. et al. High-efficiency production of bioactive recombinant human fibroblast growth factor 18 in Escherichia coli and its effects on hair follicle growth. Appl Microbiol Biotechnol. 2014; 98(2): 695-704.
